# Cultural dynamics in entrepreneurial education: Barriers, perceptions, and transformation pathways

**DOI:** 10.12688/f1000research.178134.2

**Published:** 2026-04-16

**Authors:** Alejandro Valencia-Arias, Ada Gallegos, Jackeline Valencia, Ledy Gómez-Bayona, Rutsmy Angel Manuel Gallegos Pacheco

**Affiliations:** 1 Instituto de Investigación de Estudios de la Mujer, Universidad Ricardo Palma, Santiago de Surco, 15039, Peru; 2 Vicerrectoría de Investigación y postgrado, Universidad de Los Lagos, Osorno, Los Lagos Region, 5290000, Chile; 3 Coordinación de Investigación, Institucion Universitaria Marco Fidel Suarez, Medellín, Antioquia, 50010, Colombia; 4 Enfermería, Universidad Nacional de Educacion Enrique Guzman y Valle, Lima District, Lima Region, 15453, Peru

**Keywords:** Women's entrepreneurship education, cultural barriers, female leadership, gender equality, social transformation

## Abstract

Women’s entrepreneurial education has been increasingly recognized as a mechanism for promoting gender equality and economic participation in rural contexts. However, persistent cultural, symbolic, and structural barriers continue to limit women’s access to training, leadership opportunities, and innovation ecosystems, particularly in Latin America.

**Methods:**

A systematic review was conducted following the PRISMA 2020 guidelines. Searches were performed in Scopus and Web of Science between January and September 2025. Studies published between 2015 and 2025 in English or Spanish were included if they addressed women’s entrepreneurial education, leadership, or cultural barriers in rural or comparable contexts. A total of 842 records were identified, and after screening and eligibility assessment, 129 studies were included in the qualitative synthesis and 45 in the in-depth analysis.

**Results:**

The findings indicate that institutional constraints, educational inequalities, and persistent gender stereotypes are the most frequently reported barriers. Facilitating factors include sociocultural support, visible role models, inclusive educational programs, and gender-oriented public policies. Women’s leadership is commonly described as collaborative and community-oriented, with documented associations to entrepreneurial self-efficacy, economic participation, and community resilience.

**Conclusions:**

Entrepreneurial education functions as a mediating mechanism within rural ecosystems, transforming structural and symbolic barriers into opportunities for empowerment and leadership. These findings contribute to a more comprehensive understanding of the cultural and institutional conditions shaping women’s entrepreneurial education and leadership in rural contexts.

## 1. Introduction

Women’s entrepreneurial education has been identified as a fundamental component of sustainable development, promoting economic autonomy, leadership, and the active participation of women in the social and productive transformation of communities. In rural contexts, the scope of this educational approach extends beyond the mere establishment of business enterprises. It encompasses the acquisition of competencies, principles, and mindsets that fortify the capacity to generate innovative solutions, adapt to environmental change, and spearhead local development initiatives. Consequently, it is configured as a strategic axis for gender equality, contributing to the closure of gaps in access to education, employment, and productive resources ( Miran & Gültekin, 2024; Sheena, n.d.). Nevertheless, the consolidation of women’s entrepreneurial education is confronted by numerous cultural barriers that impede women’s participation and leadership. These include gender stereotypes, patriarchal structures, the sexual division of labour, and the low value placed on women’s knowledge in rural environments.

These conditions have been shown to restrict the development of entrepreneurial skills and diminish the visibility of women as agents of change ( Christodoulou et al., 2024; Nevi et al., 2025). This is consistent with research demonstrating the persistence of social and cultural biases affecting entrepreneurial motivations, opportunities, and strategies in different contexts. The analysis of this phenomenon from a rural perspective facilitates comprehension of the manner in which cultural dynamics influence the sustainability of productive projects and the social transformation of communities. Within this framework, entrepreneurial education is aligned with the Sustainable Development Goals, particularly SDG 5 (Gender Equality) and SDG 8 (Decent Work and Economic Growth), as well as with national policies aimed at inclusion, empowerment, and territorial equity. This approach underscores the necessity to implement educational and leadership strategies that address sociocultural barriers, empower women’s innovation, and fortify rural entrepreneurial ecosystems ( Christodoulou et al., 2024; Nevi et al., 2025).

The importance of entrepreneurial education for women in achieving sustainable development and reducing inequalities is widely acknowledged. However, a knowledge gap persists regarding the limited integration of cultural and leadership dimensions in rural contexts. The extant literature addresses the role of women in entrepreneurial processes in a fragmented way, without sufficiently integrating the sociocultural factors that influence their participation and the dynamics of community leadership. This disconnection gives rise to lacunae in the comprehension of cultural structures, belief systems, and social mores that influence the consolidation of projects led by rural women, which curtails the formulation of policies and programs tailored to their realities ( Naguib, 2024; Abd El Basset et al., 2024). Empirical evidence demonstrates that the underrepresentation of women in rural entrepreneurship is associated with cultural and symbolic factors that perpetuate unequal power relations.

Patriarchal structures continue to exert a significant influence on the social and economic roles of men and women, thereby restricting women’s autonomy and decision-making power. Furthermore, the sexual division of labour and the undervaluation of knowledge generated by women serve to reinforce their exclusion from economic and political leadership positions. In many cases, the phenomenon of women’s entrepreneurship is perceived as an extension of domestic work or a secondary activity, thereby reinforcing the idea of a subordinate role in local economic development ( Miralam et al., 2025; Ahmed et al., 2025).

Moreover, a paucity of integration has been identified among educational, social, and economic approaches aimed at enhancing women’s participation in rural areas. It is evident that entrepreneurship education programmes tend to prioritise the cultivation of technical and financial competencies. However, there is a conspicuous absence of emphasis on the symbolic barriers, socialisation patterns, and power structures that impede knowledge acquisition. This absence of coherence has the effect of limiting the creation of transformative processes that incorporate women’s leadership as a strategic component of social change ( Naguib, 2024; Miralam et al., 2025).

In order to comprehend the cultural patterns that influence women’s entrepreneurial education and the configuration of rural leadership, a comparative and contextual analysis is necessary. This analysis facilitates the identification of mechanisms of exclusion and the strategies of cultural resistance developed by women, thereby providing theoretical and empirical evidence for the re-definition of educational and community practices. The knowledge gap pertains to the limited understanding of the interactions between education, culture, and rural women’s leadership, underscoring the relevance of a study that examines these links from a transformative perspective ( Abd El Basset et al., 2024; Ahmed et al., 2025).

Entrepreneurial education has become a critical mechanism for fostering economic growth, innovation, and job creation, particularly in contexts characterized by uncertainty and structural transformations. Previous studies have highlighted that entrepreneurship contributes not only to economic development but also to social value creation and improved quality of life ( Cardella et al., 2024). In this sense, universities play a key role in promoting entrepreneurial competencies and intentions among students, positioning entrepreneurial education as a strategic priority in higher education systems.

Empirical evidence has consistently demonstrated that entrepreneurship education positively influences entrepreneurial intention by enhancing motivation, skills, and attitudes toward venture creation ( Barba-Sánchez & Atienza-Sahuquillo, 2018). This relationship is particularly relevant in knowledge-based economies, where students are expected to develop both technical and entrepreneurial competencies to respond to labor market demands.

Recent studies have also emphasized the importance of contextual and individual factors in shaping entrepreneurial intentions. For instance, the COVID-19 pandemic has revealed how psychological resources such as optimism and proactivity can influence entrepreneurial intention in adverse environments ( Cardella et al., 2024). Similarly, emerging research highlights the role of sustainability-oriented variables, such as ecological awareness, and gender differences in shaping entrepreneurial intentions, indicating that entrepreneurial education must adapt to evolving societal and environmental challenges ( Atienza-Barba et al., 2025).

Despite these advances, there is still a need for integrative approaches that consider cultural dynamics as a central element in entrepreneurial education. This study responds to this need by examining how cultural barriers, perceptions, and transformation strategies interact to shape entrepreneurial education processes.

Despite the growing body of literature on entrepreneurial education, existing studies have predominantly focused on general pedagogical approaches, often overlooking the role of cultural dynamics as a structuring factor influencing learning processes and entrepreneurial intentions. In particular, there is a limited understanding of how cultural barriers interact with individual perceptions and institutional conditions to shape entrepreneurial education outcomes.

Furthermore, prior research has tended to analyze these dimensions in isolation, lacking an integrative perspective that connects barriers, perceptions, and transformation strategies within a unified analytical framework. Therefore, restricts the development of context-sensitive educational interventions, especially in environments where cultural norms strongly influence participation and engagement.

To address these gaps, this study offers a comprehensive analysis of cultural influences in entrepreneurial education by integrating three key dimensions: barriers, perceptions, and transformation pathways. The study contributes to the literature by proposing a multidimensional perspective that not only identifies constraints but also highlights actionable strategies for educational transformation. In doing so, it advances current knowledge by linking cultural context with practical implications for the design of more inclusive and effective entrepreneurial education programs.

The objective of this research is to identify the cultural barriers and transformative factors that influence entrepreneurial education and women’s leadership in rural Latin American contexts. In order to achieve this objective, a series of questions have been devised to guide understanding of the cultural barriers and transformative factors associated with women’s leadership in rural contexts.

1. What characteristics define the most frequent leadership models among rural women according to the academic literature?
2. What social, cultural, or institutional factors have facilitated the emergence of women’s leadership in rural areas?
3. What barriers have limited the recognition or effective participation of rural women in community or political leadership spaces?
4. What impacts have been documented in communities where rural women hold active leadership roles?
5. What approaches or programs have been implemented to strengthen leadership capacities in rural women?

## 2. Methodology

The research employed the PRISMA 2020 (Preferred Reporting Items for Systematic Reviews and Meta-Analyses) methodology as a guide for the systematic review, in order to ensure transparency, rigour, and reproducibility in the search, selection, and synthesis of scientific evidence. This methodological approach provides a structured framework that facilitates the identification, evaluation, and coherent presentation of results obtained from verified academic sources, guaranteeing the traceability of the entire process. The application of the PRISMA model enabled the analysis of studies related to cultural barriers, entrepreneurial education, and female leadership in rural Latin American contexts, following the four phases defined by the protocol: identification, screening, eligibility, and inclusion. These phases enabled the refinement of the information and the final selection of the most relevant articles ( Page et al., 2021).

### 2.1 Eligibility criteria

The selection of studies included in the review was based on eligibility criteria defined to ensure relevance, quality, and coherence with the research objectives. The inclusion criteria encompassed peer-reviewed articles published between 2015 and 2025, written in English or Spanish, that addressed topics related to entrepreneurial education, women’s entrepreneurship, leadership, and cultural barriers. The study incorporated a range of research methodologies, including qualitative, quantitative, and systematic review approaches, with the overarching objective being the presentation of empirical or theoretical evidence on women’s participation in entrepreneurial training, rural leadership, or social transformation. The selection prioritised studies analysing the relationship between education, culture, and gender in local development contexts, with a particular focus on Latin America, Africa, and Asia, in order to identify global patterns and regional specificities. The parameters for the literature search and evaluation were established with the objective of ensuring the validity and traceability of the results.

It is important to note that only studies from academic databases recognised for their rigour and scientific visibility were included in the study. This ensured the reliability of the evidence used. The selected documents addressed the phenomenon from social, cultural, and institutional perspectives, supported by consistent theoretical frameworks and verifiable methodologies. A range of studies exploring the intersection of gender, education and economic development were also considered, with a particular emphasis on how cultural factors influence leadership and entrepreneurship opportunities for rural women. The exclusion process was executed in three successive phases.

In the initial phase of the study, articles containing indexing errors or duplicate records were eliminated. In the second instance, those without full-text access were discarded due to the impossibility of analysing their methodological content. In the third study, an exclusion criterion was applied. This criterion was based on thematic relevance, theoretical depth, and the absence of novel contributions compared to other studies. The implementation of these methodologies resulted in the creation of a comprehensive and high-quality set of studies, thereby ensuring a robust methodological foundation for the analysis of cultural barriers and transformative factors in women’s entrepreneurial education across diverse global regions.

### 2.2 Sources of information

The systematic review was based on two highly visible and internationally recognised academic databases: Scopus and Web of Science. The selection of these platforms was based on their thematic and geographic coverage, as well as their relevance to scientific production in the areas of social sciences, education, and business studies. It is important to note that both of these databases are regarded as benchmarks in the evaluation of scientific knowledge. In addition, they provide access to publications that have been indexed in peer-reviewed journals. This ensures the reliability, traceability and consistency of the retrieved information. The amalgamation of these two sources served to enhance the representativeness of the included studies, thereby mitigating biases that might have arisen from editorial concentration. This approach ensured a more comprehensive coverage of regions and disciplines ( Asubiaro, Onaolapo, & Mills, 2024). The search was conducted between January and September 2025, incorporating original articles, systematic reviews, and academic chapters that contributed empirical or theoretical evidence on women’s entrepreneurial education, rural leadership, and cultural barriers.

The process entailed a comprehensive review of the available records in each database, with pre-established inclusion and exclusion criteria being applied to ensure consistency and methodological rigor. Scopus and Web of Science both offer comprehensive metadata, including authorship, year of publication, country, institutional affiliation, subject area, number of citations, and document type. These elements enabled the organisation and classification of results according to scientific and geographical variables. This configuration enabled the refinement of records and the identification of emerging trends in literature. Publications in journals that have been ranked in the Q1 and Q2 quartiles of the Scimago Journal Rank (SJR) were prioritised, thus ensuring the selection of high-impact studies that demonstrate methodological quality. The search was complemented by a manual check in Google Scholar, the aim of which was to locate grey literature and recent works related to rural women’s leadership and cultural transformation.

### 2.3 Search strategy

A specific search equation was established for each database, formulated according to the inclusion criteria and guidelines of the PRISMA 2020 model. In Scopus, the equation employed was TITLE (“entrepreneurial education” OR “entrepreneurship education”) AND TITLE-ABS-KEY (“women” OR “female”), while in Web of Science, its syntax was adapted using the equivalent structure TS=(“entrepreneurial education” OR “entrepreneurship education”) AND TS=(“women” OR “female”). The derivation of both equations was undertaken in accordance with the stipulated inclusion criteria, with the objective of ensuring thematic precision and methodological consistency.

The keywords were articulated using Boolean operators AND and OR, supplemented with truncation and related terms such as “female entrepreneurship,” “cultural barriers,” “rural leadership,” “gender equality,” and “empowerment strategies.” This formulation enabled the retrieval of relevant and contemporary studies on female entrepreneurship education, rural leadership, and cultural barriers. The initial search generated a large number of records, which were subsequently refined according to the defined exclusion criteria, ensuring the coherence and quality of the final corpus for analysis.

### 2.4 Selection process

**Figure 1.**
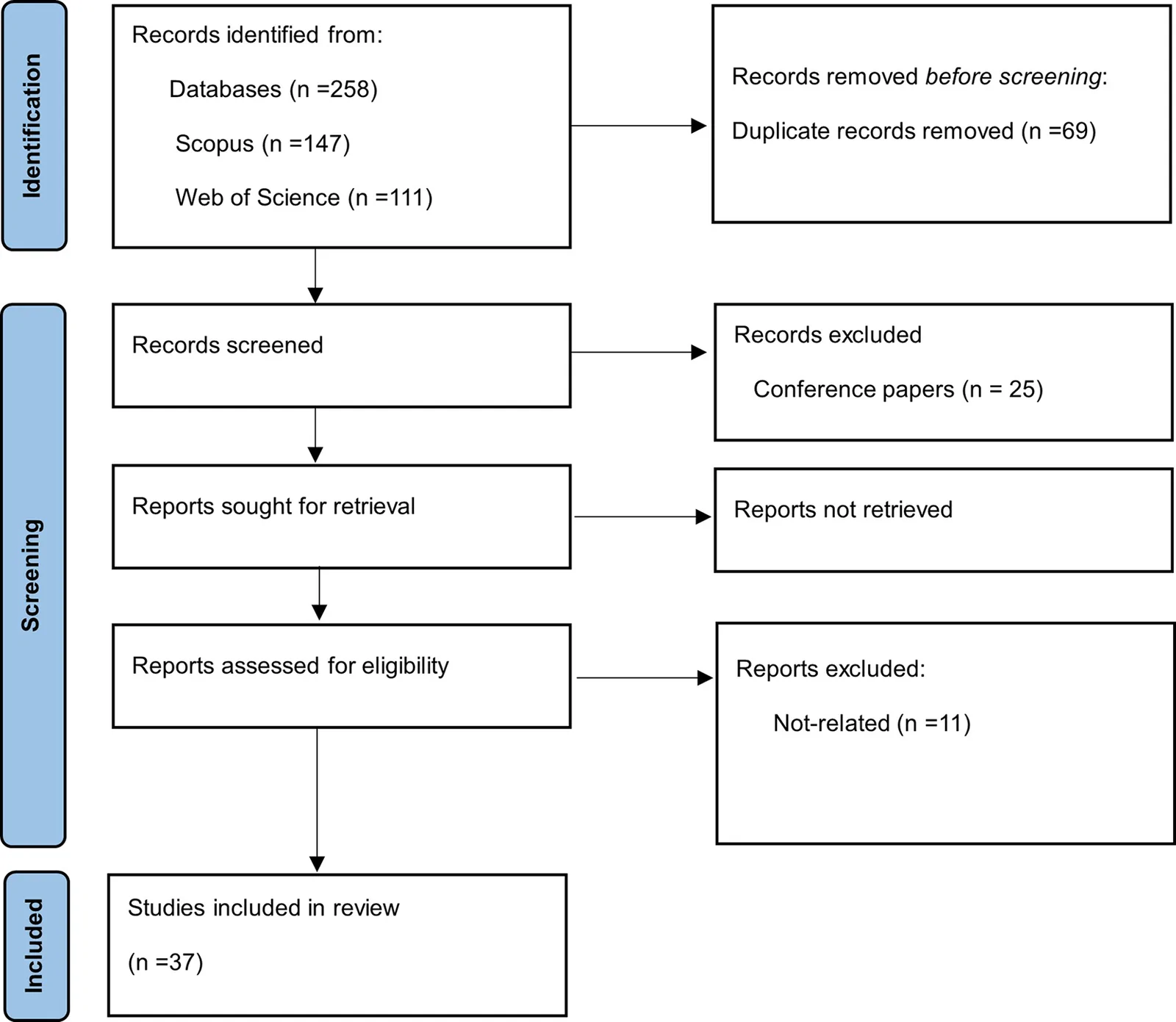
PRISMA flowchart. Created by the author using data from Scopus and Web of Science.

The selection of studies was carried out in three consecutive stages, following a systematic procedure designed to guarantee the relevance and quality of the final corpus. In the initial phase, the titles and abstracts were subjected to a review process to ensure thematic concordance and to remove redundancies across the various databases. In the subsequent stage, the complete texts of the articles that had been selected at the first stage were analysed. The methodological consistency and coherence of these articles with the inclusion criteria that had been defined was assessed. In the third stage, the final selection was made based on the theoretical relevance and scientific rigour of each document. The review process was overseen by two researchers, who conducted independent reviews of each record to minimise the potential for bias. Disagreements were resolved through consensus. The entire procedure is represented in Figure 1, corresponding to the PRISMA 2020 flowchart.

A total of 842 records were identified from the Scopus (472) and Web of Science (370) databases. A further 18 records were incorporated into the study through a manual search of Google Scholar and a review of bibliographic references. During the preliminary data cleansing stage, 112 duplicate records were eliminated, resulting in a total of 748 unique studies available for review. In the screening phase, titles and abstracts were examined, and 421 articles were excluded due to a lack of relevance to women’s entrepreneurial education, leadership, or cultural barriers. In the eligibility phase, 327 full texts were analysed, and 198 were excluded for not meeting the inclusion criteria, either due to indexing errors, restricted access, or a lack of focus on rural women’s leadership. In the final analysis, 129 studies were included in the qualitative synthesis and 45 articles in the detailed analysis of results. The complete process is represented in Figure 1, corresponding to the PRISMA 2020 flow diagram.

### 2.5 Data processing

Microsoft Excel was utilised as the primary instrument for the systematic documentation, organisation, and structuring of the data obtained during the review process. This approach ensured the implementation of a structured procedure that facilitated the traceability of the analytical process. The data were coded based on precise variables, including author, year of publication, country, type of study, methodological approach, main results, and identified barriers, which allowed for consistency in the classification and control of the consulted sources. The information was organised into pivot tables that facilitated comparative analysis and the generation of descriptive graphs aimed at visualising trends. The findings were grouped into four thematic areas, namely cultural barriers, transformation strategies, entrepreneurial education, and women’s leadership, which facilitated the identification of recurring patterns, knowledge gaps, and emerging lines of research related to women’s participation in entrepreneurship and rural leadership processes.

Although specialized qualitative data analysis software such as ATLAS.ti or NVivo is commonly recommended for coding and thematic synthesis in systematic literature reviews, the present study adopted a structured manual coding approach supported by spreadsheet-based tools. Specifically, pivot tables were used not only for data organization but also to facilitate systematic categorization, comparison across studies, and the identification of thematic patterns.

This approach ensured analytical rigor by applying predefined coding criteria, consistent classification procedures, and iterative validation of categories throughout the review process. Moreover, the use of spreadsheet-based analysis enabled transparent data handling, traceability of coding decisions, and reproducibility of results, as all transformations and categorizations were explicitly documented.

It is important to note that previous systematic reviews have successfully employed similar approaches when dealing with structured bibliographic data, particularly in studies focused on thematic classification and comparative analysis. Therefore, while advanced qualitative software may enhance analytical depth in certain contexts, the selected methodology is appropriate for the objectives of this study and ensures sufficient rigor, transparency, and replicability.

### 2.6 Risk of bias

The risk of bias was assessed by identifying methodological limitations and potential publication biases in the selected studies. This was achieved by considering the transparency of procedures, the diversity of sources, geographical balance, and the clarity of results presentation. This process enabled the determination of the level of reliability of the analysed evidence. The process incorporated a qualitative evaluation criterion aimed at weighing the methodological and theoretical quality of the articles, classifying them as having high, medium, or low reliability according to their consistency and analytical rigor. The potential biases arising from the use of specific databases, the selection of keywords, and the limitations in the reporting of results were also identified, as these elements have the capacity to affect the representativeness of the final corpus. The results are summarised in Figure 1, corresponding to the PRISMA 2020 flow diagram.

## 3. Results

The results were organised according to the five research questions in order to comprehensively examine cultural barriers, transformative factors, and the dynamics of women’s leadership in rural settings. The analysis of the empirical and theoretical evidence collected allowed for the identification of common patterns across regions, along with contrasts stemming from the social, institutional and cultural conditions of each context. Emerging trends have been observed, indicating an evolution in the leadership of rural women in the face of structural and symbolic limitations that constrain their participation. Table 1 provides a synopsis of the selected studies that underpinned the comparative analysis and interpretation of the findings.

**Table 1.** Studies included in the research. Prepared by the author using data from Scopus and Web of Science.

Title	Authors (Year)	Main Themes	Methodology	Sample/Context	Key Findings/Conclusions
Analyzing the role of gender in entrepreneurship education and economic success in developing nations: the case of Colombia	de la Puente Pacheco et al., (2025)	Gender in entrepreneurship education and business success	Quantitative (Chi-square, t-tests, Regression analysis)	89 participants (Barranquilla, Colombia)	Gender-specific differences in program access; positive relationship between education and business success for both genders.
Creating Shared Value in Banking by Offering Entrepreneurship Education to Female Entrepreneurs	Taskin S et al., Y 2023	Creating Shared Value (CSV) in female-centered banking and entrepreneurship education	Qualitative (Case study)	Female entrepreneurs (City Alo, Bangladesh)	Entrepreneurship education fosters client prosperity and addresses socio-economic challenges in women’s entrepreneurship.
Does entrepreneurship education in China develop entrepreneurial intention? the role of self-efficacy and experience	Xu J et al., 2023	Entrepreneurship education, self-efficacy, and entrepreneurial intention	Quantitative (Structural Equation Modeling)	243 participants (China, female = 40.3%)	Entrepreneurship education has a significant positive impact on entrepreneurial intention; self-efficacy mediates this effect.
Entrepreneurship education and entrepreneurial intention: Do female students benefit?	Westhead and Solesvik (2016)	Links between entrepreneurship education, alertness, risk-taking, and gender	Quantitative (Hierarchical regression)	Business and engineering students (Unspecified university)	Female students in entrepreneurship education report lower intensity of entrepreneurial intention, but those citing alertness skills have higher intentions.
Entrepreneurship education in TVET institutions and entrepreneurial intentions of female students in Ghana: the social support factor	Padi A et al., 2022	Impact of TVET entrepreneurship education on female students’ intentions	Mixed-methods (Quantitative survey + Interviews)	376 female students (Ghana)	Entrepreneurship education positively influences female students’ entrepreneurial intentions, stronger with social support systems.
Evaluating the Role of Entrepreneurship Education in Shaping Female College Students’ Career Choices in China: A Multidimensional Analysis	Sun SL et al., 2025	Gender, entrepreneurship education, and career choices	Quantitative (Regression analysis)	24,508 female students (China, across 31 provinces)	Courses and competitions are critical for female students' entrepreneurial career choices; policy initiatives have a limited effect.
Financial Literacy as a Key to Entrepreneurship Education: A Multi-Case Study Exploring Diversity and Inclusion	Medina-Vidal A et al., 2023	Financial literacy and its impact on entrepreneurial education	Mixed-methods (Focus groups + Questionnaires)	Young Mexican students (17–24 years old, five cities)	Financial literacy correlates with better financial behaviors; critical thinking enhances financial decision-making, reducing the gender gap.
GENDER ROLES IN ENTREPRENEURSHIP EDUCATION TO SOCIAL ENTREPRENEURIAL INTENTIONS IN VIETNAM	Lan et al. (2023)	Gender roles in social entrepreneurship education and intentions	Quantitative (Partial least squares SEM)	811 students (Vietnam)	Significant gender differences in social entrepreneurship intentions; entrepreneurship education has varying effects by gender.
Gendered Entrepreneurship Education and the Fear of Failure	Guelich, U. (2022)	Gendered entrepreneurship education and the fear of failure	Quantitative (Survey + SEM)	1,668 entrepreneurs (Thailand)	Fear of failure impacts innovation, but knowing other entrepreneurs reduces its effect, especially for women.
Impact of Innovation and Entrepreneurship Education in a University Under Personality Psychology Education Concept on Talent Training and Cultural Diversity of New Entrepreneurs	Li et al., (2021)	Innovation and entrepreneurship education in universities	Mixed-methods (Surveys + Interviews)	University students (China, different majors)	Innovation dimension scores higher in students, with female students showing slightly higher challenge spirit; social and cultural diversity influence entrepreneurship outcomes.
Improving the Entrepreneurial Competence of College Social Entrepreneurs: Digital Government Building, Entrepreneurship Education, and Entrepreneurial Cognition	Xiang et al. (2022)	Entrepreneurship education, digital government, and gender differences	Quantitative (Regression analysis)	20,134 female students (31 provinces, China)	EE and digital government building positively influence female social entrepreneurs’ competence; gender differences observed.
Overcoming Gender Gaps in Entrepreneurship Education and Training	Pimpa, N. (2021)	Financial literacy and its impact on entrepreneurship education	Mixed-methods (Focus groups + Questionnaires)	17–24-year-old students (Mexico, five cities)	Critical thinking positively correlates with financial behaviour; financial literacy reduces the gender gap in financial decision-making.
The effect of entrepreneurship education on the entrepreneurial intention of different college students: Gender, household registration, school type, and poverty status	Deng & Wang (2023)	Gender roles in social entrepreneurship education and social entrepreneurship intention	Quantitative (Partial least squares SEM)	811 students (Vietnam)	Significant gender differences in social entrepreneurship intentions; EE’s impact on gender is unclear.
The Impact of Entrepreneurial Self-Efficacy and Entrepreneurship on Entrepreneurial Intention: Entrepreneurial Attitude as a Mediator and Entrepreneurship Education Having a Moderate Effect	Ye & Kang (2025)	Gendered entrepreneurship education and the fear of failure	Quantitative (Survey + SEM)	1,668 entrepreneurs (Thailand)	Fear of failure impacts innovation, but knowing other entrepreneurs reduces its effect, especially for women.
Unveiling the role of entrepreneurship education on green entrepreneurial intentions among business students: gender as a moderator	Makuya & Changalima (2024)	Impact of entrepreneurship education on green entrepreneurial intentions	Quantitative (PLS-SEM)	204 business students (Tanzania)	EE positively impacts green entrepreneurial intentions; gender does not moderate this relationship despite stronger effects in males.
College students’ entrepreneurship education path and management strategy of start-up enterprises using causal attribution theory	Liu et al., (2025)	Entrepreneurship education, digital government, and gender differences	Quantitative (Regression analysis)	20,134 female students (31 provinces, China)	EE and digital government building positively influence female social entrepreneurs’ competence; gender differences observed.
Educators and students in entrepreneurship education are challenging the “think entrepreneur–think male” paradigm	Stoker et al., (2025)	Gender issues in entrepreneurship education	Qualitative (Interviews)	32 students and educators (Netherlands)	Gendered constructs in EE are changing; the “think entrepreneur–think male” paradigm is being challenged.
Entrepreneurial, economic, and social well-being outcomes from an RCT of a youth entrepreneurship education intervention among native American adolescents	Tingey et al., (2020)	Entrepreneurship education, economic empowerment, and social well-being	Quantitative (RCT) + Qualitative (Focus groups)	394 Native American youth (USA)	Significant improvements in entrepreneurship knowledge and economic confidence among Native American youth; strong impact on social well-being.
Entrepreneurship Education among University Students as a Predictor of Female Entrepreneurial Undertakings	Vukmirović, V. (2019)	Female entrepreneurship and education	Conceptual (Literature review)	Female entrepreneurs (Global)	Gender-specific barriers to female entrepreneurship; proposes a conceptual model to address these barriers.
Entrepreneurship education and disability: An experience at a Spanish university	Muñoz et al., (2019)	Entrepreneurship education for disabled students	Quantitative (ANOVA)	50 students (Spain, with and without disabilities)	No significant differences between disabled and non-disabled students in terms of entrepreneurial attitudes after EE.
Entrepreneurship Education for Women—European Policy Examples of Neoliberal Feminism?	Berggren, C 2020	Neoliberal feminism, gender in entrepreneurship education	Policy analysis (Neoliberal feminism perspective)	European Union policies (2004–2018)	The focus of EE policies is more about ideological education than promoting women’s self-employment.
Exploration and Practice of Maker Education Mode in Innovation and Entrepreneurship Education	Yang, Y. (2020)	Impact of entrepreneurial psychological quality and maker education	Quantitative (Survey + SPSS analysis)	20,000+ students (China, different majors)	EE and maker education influence entrepreneurial competence; family economy, sports, and grades impact students’ innovation and entrepreneurship abilities.
Gender-sensitive vocational and entrepreneurship education: addressing poverty for Caribbean women	Bahaw et al., 2025	Gendered poverty, entrepreneurship education in TVET	Qualitative (Literature review)	Women in the Caribbean	Integrating EE into TVET can empower women, but gender-sensitive practices are critical to reducing barriers and enhancing opportunities.
Generative artificial intelligence in entrepreneurship education enhances entrepreneurial intention through self-efficacy and university support	Xie, Y; Wang, S 2025	Generative AI in entrepreneurship education	Quantitative (SEM)	346 students (China)	Generative AI enhances entrepreneurial self-efficacy and intention, with university support strengthening the effects.
How does entrepreneurial curiosity stimulate new venture ideas among Chinese undergraduates? The mediating role of promotion focus and the moderating role of entrepreneurial education	Li, C; Hu, R 2025	Entrepreneurial curiosity and new venture ideas	Quantitative (PLS-SEM)	650 undergraduates (China)	I-type entrepreneurial curiosity positively affects new venture ideas; entrepreneurial education moderates this effect.
Impact of attitude towards entrepreneurship education and role models on entrepreneurial intention	Amofah & Saladrigues (2022)	Entrepreneurial intention, role models, gender, and EE	Quantitative (SEM, Multi-group analysis)	216 students (Spain)	Gender differences in entrepreneurial intention; the relationship between PSE and entrepreneurial competence is stronger for males.
Impact of personality traits and entrepreneurship education on entrepreneurial intentions of business and engineering students	Vodă & Florea (2019)	Personality traits, entrepreneurial education, and entrepreneurial intention	Quantitative (Multivariate logistic regression)	270 students (Romania)	Locus of control, need for achievement, and entrepreneurial education influence venture creation; males more inclined towards entrepreneurship.
Role modeling as a pedagogical strategy in entrepreneurship education for women and girls: An interactive model of transformational learning	Oppedisano & Laird (2006)	Role modeling in entrepreneurship education for women	Qualitative (Interviews, case study)	Undergraduate students (US, multidisciplinary course)	Role models positively influence female students’ perceptions of entrepreneurship and their career aspirations.
Teacher-student empathic relationship shaping: an elimination mechanism for psychological segmentations in entrepreneurship education	Yi et al., (2025)	Teacher-student empathy, psychological segmentation, and EE	Quantitative (Experiments, SEM)	424 students (China)	Empathic teacher-student relationships enhance entrepreneurial confidence; cognitive and affective empathy play significant roles.
Teaching through television: Experimental evidence on entrepreneurship education in Tanzania	Bjorvatn et al., (2020)	Edutainment, entrepreneurship education, and school performance	Experimental (RCT, Field experiment)	2,000 secondary school students (Tanzania)	Edutainment show increased interest in entrepreneurship, particularly among females, but had negative effects on school performance.
The analysis of the effect of entrepreneurship education, perceived desirability, and entrepreneurial self-efficacy on university students’ entrepreneurial intention	Suratno et al., (2019)	Entrepreneurship education, self-efficacy, perceived desirability, gender differences	Quantitative (SEM, Cross-sectional)	505 alumni (Jambi University, Indonesia)	EE positively affects entrepreneurial intention; gender differences observed, with males having stronger self-efficacy.
The Development of Inclusive Agriculture Entrepreneurship Education Ecosystems for Young Entrepreneurs in Uganda	Bullock et al., (2025)	Inclusive agricultural entrepreneurship education in Uganda	Qualitative (Discourse analysis, interviews)	16 student entrepreneurs (Uganda)	Gender and financial literacy gaps remain barriers for women; EE should be more inclusive and support financial access.
The Effect of Entrepreneurship Education on Entrepreneurial Intention: Mediation of Entrepreneurial Self-Efficacy and Moderating Model of Psychological Capital	Wang et al., (2023)	Entrepreneurship education, self-efficacy, psychological capital	Quantitative (SEM, Structural equation modeling)	757 students (Guangxi, China)	EE significantly impacts entrepreneurial intention; psychological capital moderates self-efficacy’s effect.
The Influence of Economic and Entrepreneurial Education on Perception and Attitudes towards Entrepreneurship	Ilieș et al., (2023)	Economic and entrepreneurial education, entrepreneurial intention	Quantitative (Regression analysis, PCA)	582 students (Romania)	EE and economic background significantly predict entrepreneurial intention, with students from economic backgrounds showing stronger intention.
The Re-integration of College Innovation and Entrepreneurship Education Under Young Entrepreneurs’ Enterprising Spirit and Professional Music Education	Qu et al., (2022)	Integration of innovation and entrepreneurship education with professional education	Quantitative (Survey, descriptive stats)	305 students (Music College, Xi’an, China)	Integration of IEE and professional education needs improvement; gender differences in entrepreneurship awareness.
The role of entrepreneurship education as a predictor of university students’ entrepreneurial intention	Zhang et al., (2014)	Entrepreneurship education, entrepreneurial intention, gender differences	Quantitative (Probit estimation)	494 students (10 universities, China)	Entrepreneurship education positively impacts entrepreneurial intention; gender, university type, and major significantly affect EI.
UNPACKING ENTREPRENEURIAL EDUCATION: LEARNING ACTIVITIES, STUDENTS’ GENDER, AND ATTITUDE TOWARD ENTREPRENEURSHIP	Padilla-Angulo et al., (2022)	Gender differences in entrepreneurial attitude and education	Quantitative (Survey, Post-hoc tests)	918 students (French business school)	Gender differences in entrepreneurial attitude; specific academic activities can boost EPA, with gender and academic level influencing outcomes.

**Figure 2.**
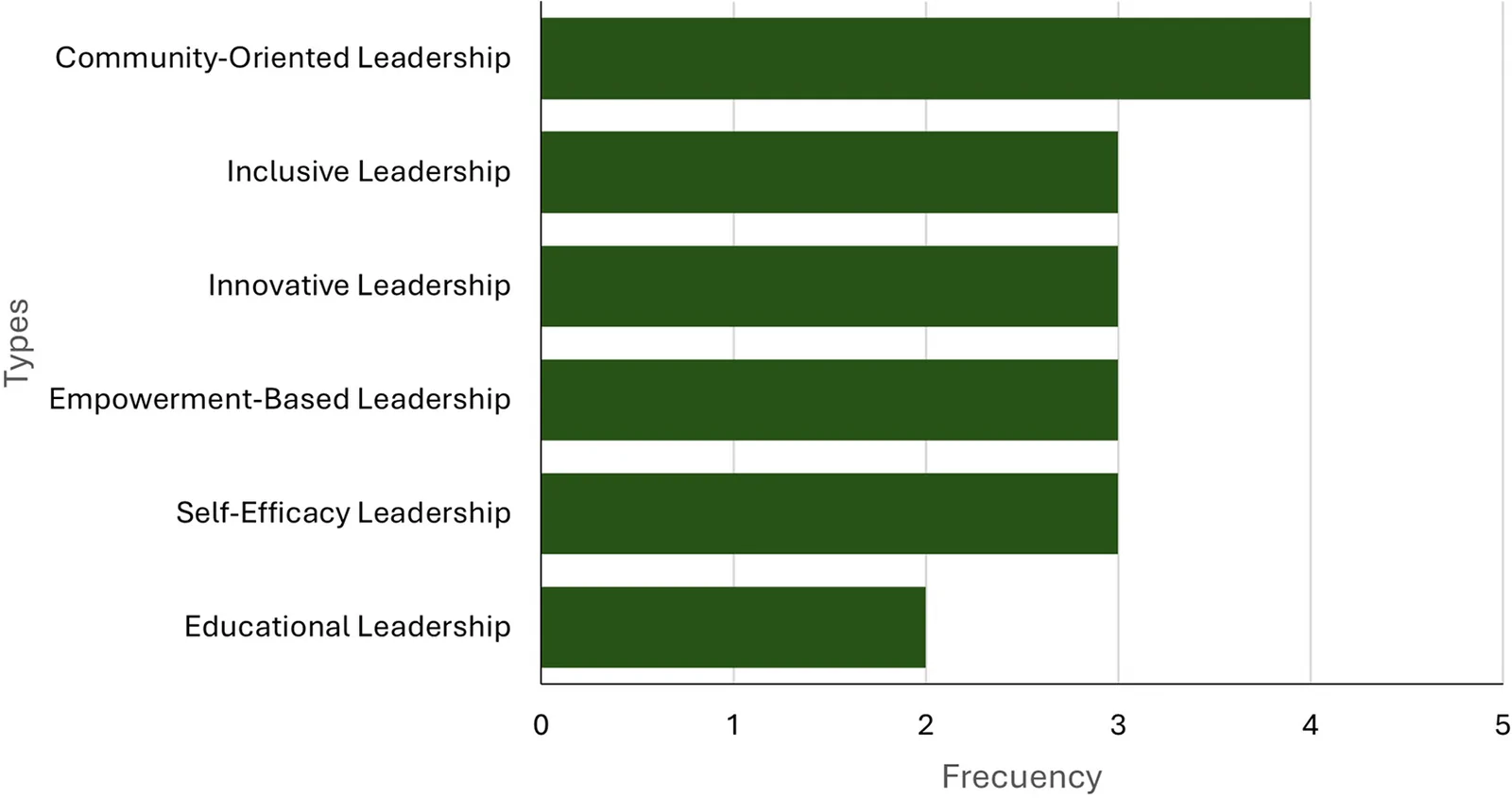
Distribution of female leadership typologies. Prepared by the author using data from Scopus and Web of Science.

The results are presented in Figure 2, which illustrates the distribution of the primary typologies of female leadership identified in the review. Community leadership is predominant, followed by inclusive, innovative, empowerment-centred, and self-efficacy-oriented approaches, while educational leadership is less frequent. This trend underscores the value of collaborative and transformational approaches in female leadership processes linked to education and social participation.

**Figure 3.**
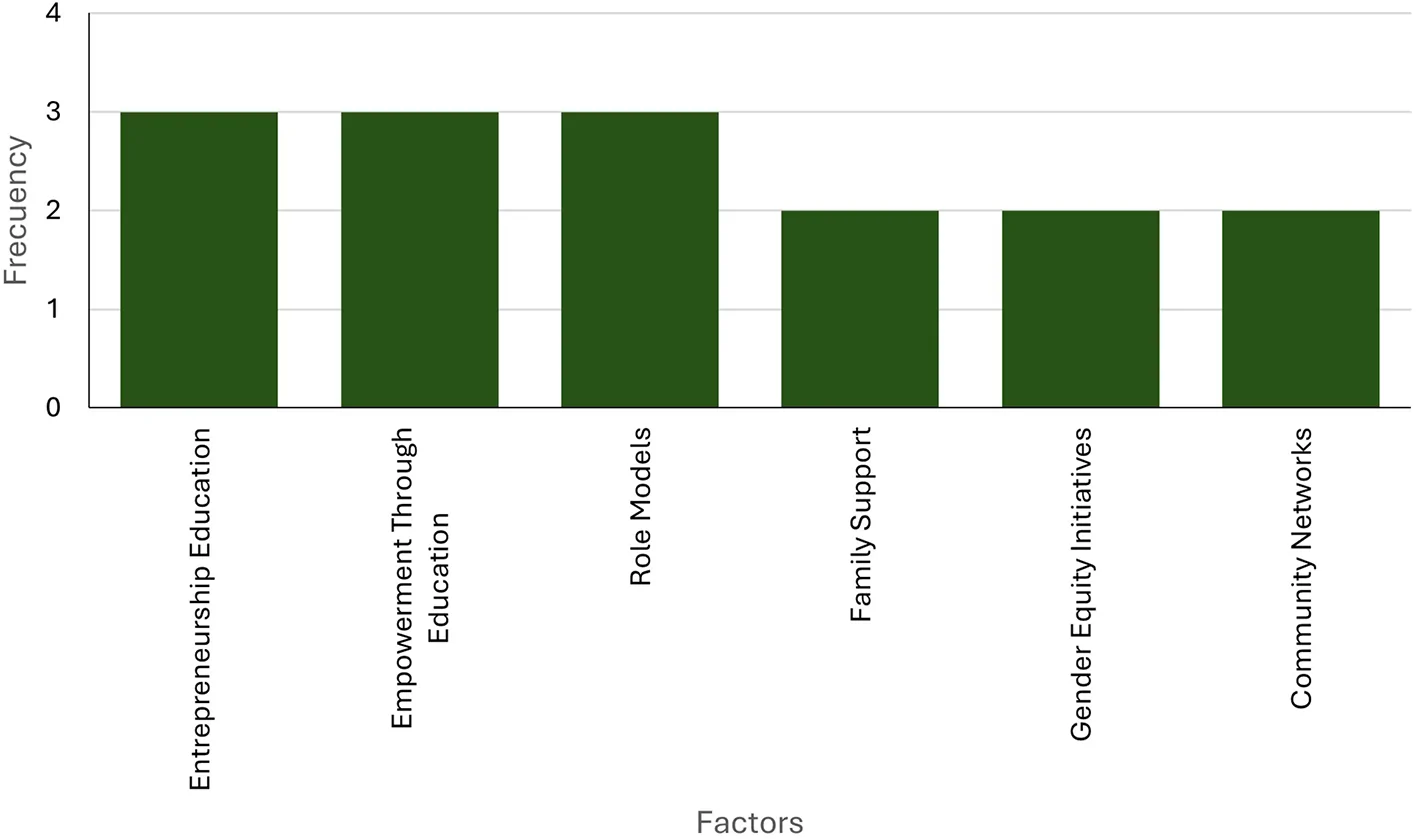
Sociocultural factors facilitating female entrepreneurship. Prepared by the author based on Scopus and Web of Science.

The results presented in Figure 3 demonstrate the distribution of the primary sociocultural factors that promote women’s entrepreneurial education. The role of entrepreneurial education, the empowerment of individuals through education, and the influence of role models are foundational, followed by family support, gender equality initiatives, and community networks. Collectively, these factors contribute to the creation of an environment conducive to the development of women’s leadership. The findings underscore the pivotal role of academic training, social support, and the visibility of role models in enhancing women’s entrepreneurial skills.

**Figure 4.**
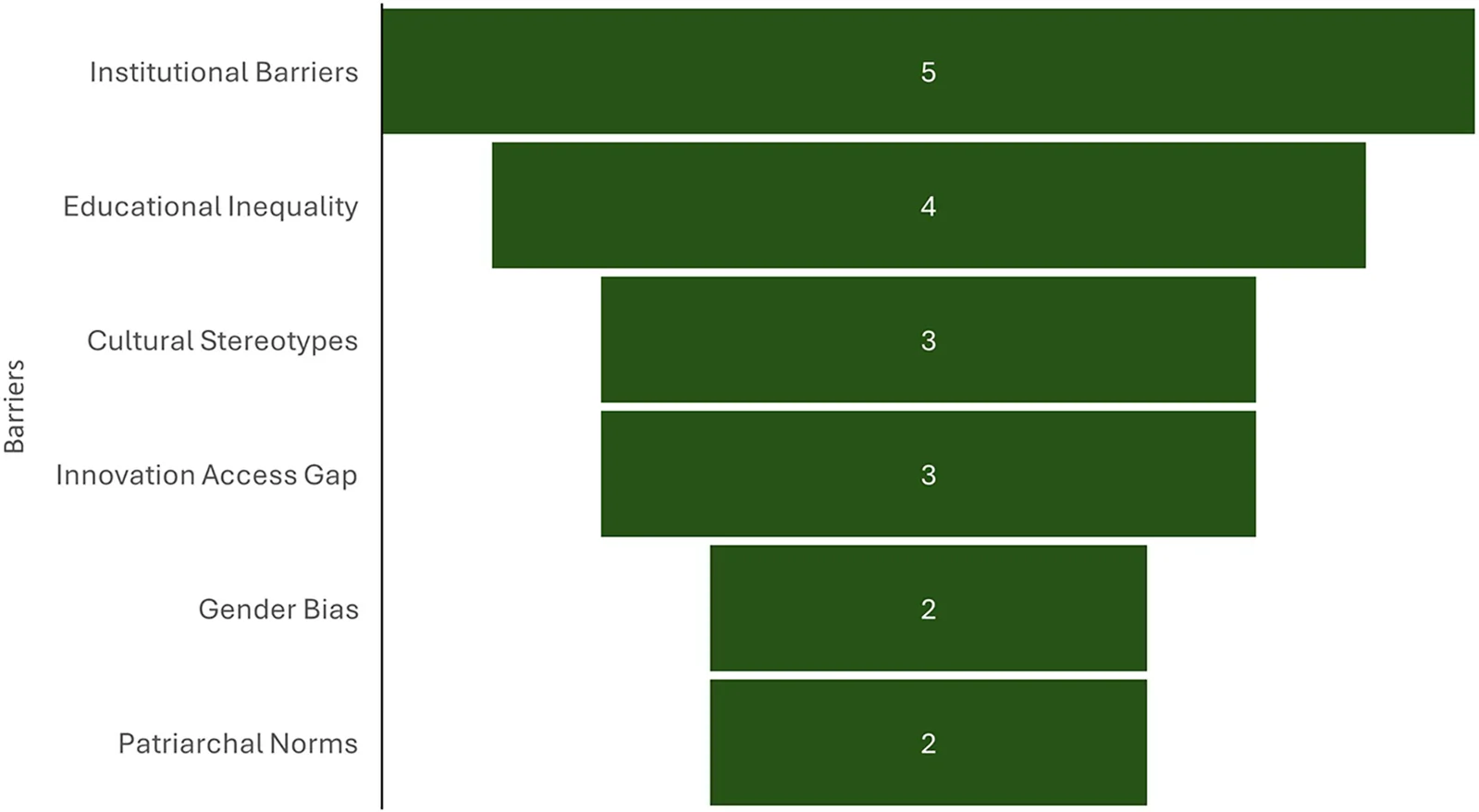
Structural and symbolic barriers in female entrepreneurial education. Prepared by the author based on Scopus and Web of Science.

The results presented in Figure 4 illustrate the distribution of the primary structural and symbolic barriers that condition women’s participation in entrepreneurial education. The prevalence of institutional barriers is particularly salient, followed by issues of educational inequality, cultural stereotypes, and disparities in access to innovation. The influence of gender bias and patriarchal norms is identified to a lesser extent, yet these factors continue to exert their influence in educational and business spheres, thereby reflecting the persistence of inequalities that hinder the development of women’s entrepreneurial leadership.

**Figure 5.**
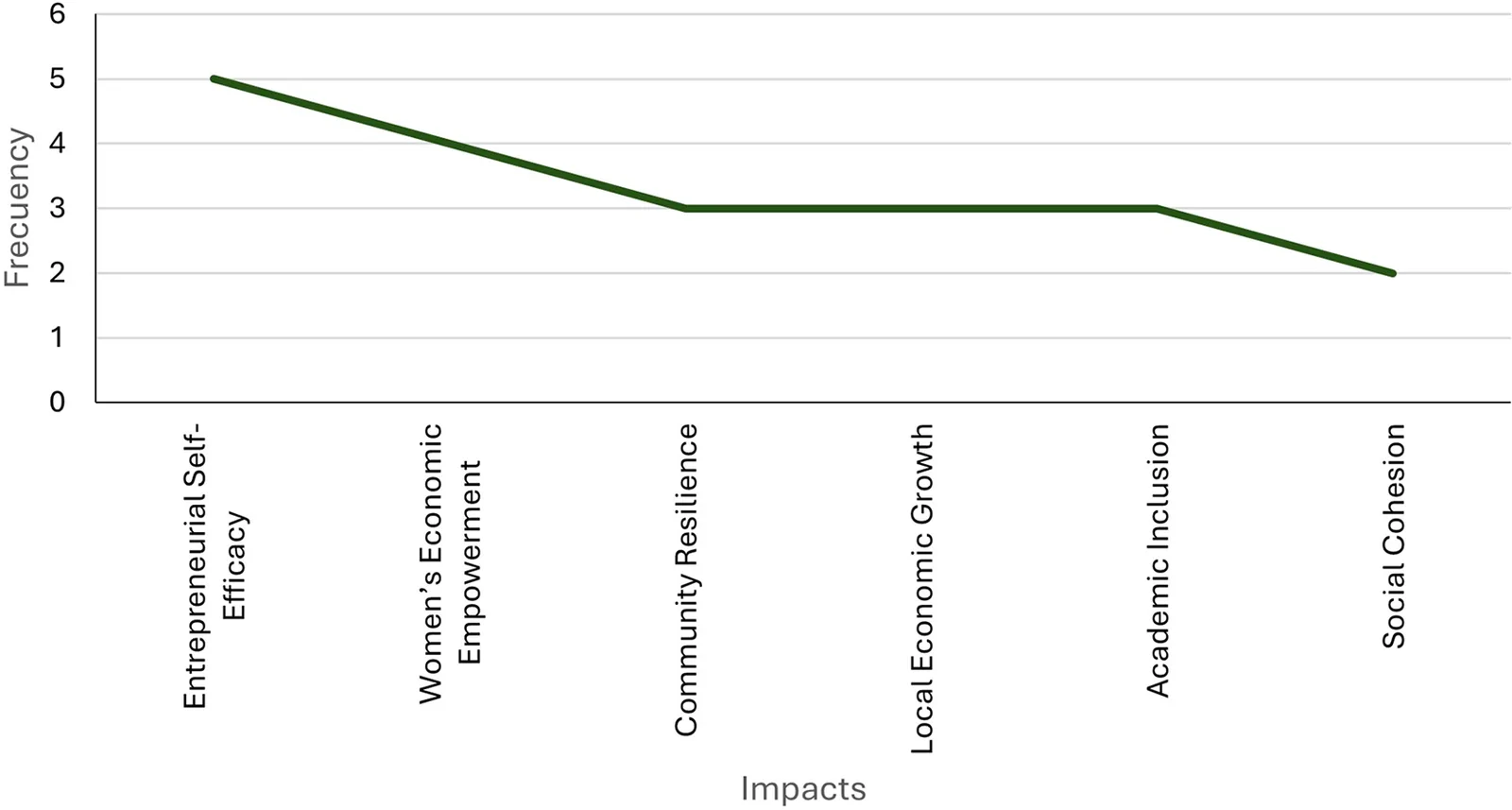
Documented community impacts on female entrepreneurial education. Prepared by the author based on Scopus and Web of Science.

The results presented in Figure 5 illustrate the distribution of the primary documented community impacts associated with women’s entrepreneurship education. It is evident that entrepreneurial self-efficacy and women’s economic empowerment are of particular significance, in addition to the enhancement of community resilience, local economic growth, and academic inclusion. These elements signify substantial progress in capacity building and opportunities. Social cohesion, an indicator of the consolidation of collective bonds and the positive impact of women’s participation in entrepreneurial ecosystems, is identified to a lesser extent.

**Figure 6.**
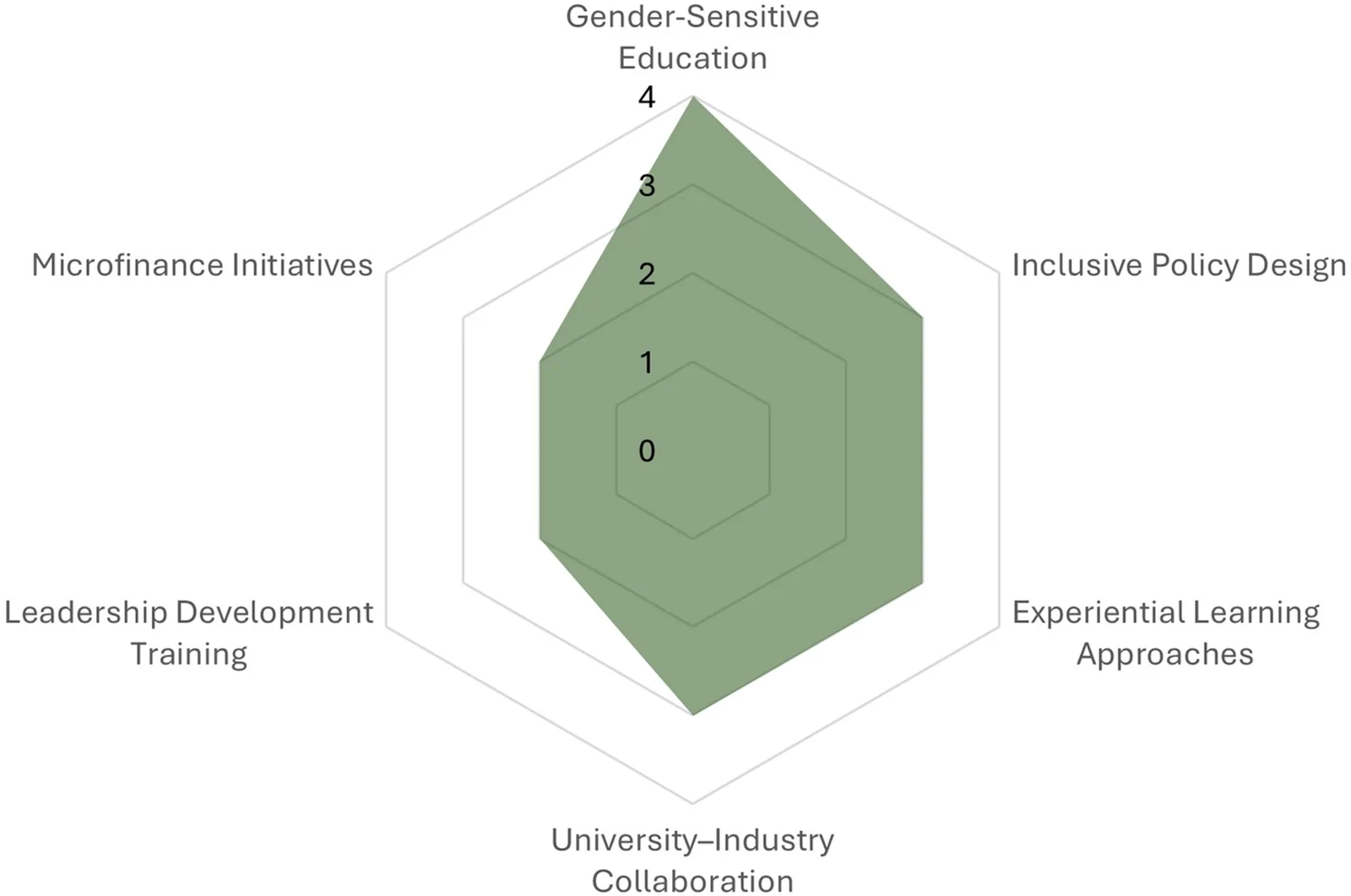
Programs and approaches for strengthening women's entrepreneurial education. Prepared by the author based on Scopus and Web of Science.

The results presented in Figure 6 illustrate the distribution of the primary programmes and strengthening approaches identified in women’s entrepreneurship education. Gender-focused education initiatives are of particular note, followed by inclusive policy designs and experiential learning approaches, which are notable for their applicability in academic and social settings. Evidence suggests that actions aimed at fostering collaboration between universities and industry are in evidence, as are leadership and microfinance training programmes. These measures contribute to reducing structural gaps and promoting women’s participation in entrepreneurship.

The results were organised according to the research questions and allowed for a comprehensive understanding of the dimensions that comprise women’s entrepreneurial education. The analysis revealed the interaction of structural, symbolic, and community factors that influence women’s leadership and participation, along with programs aimed at strengthening their capacities.

## 4. Discussion

The findings of this study can be interpreted in light of existing theoretical frameworks on entrepreneurial education and intention. In line with previous research, the results confirm that entrepreneurial education is strongly influenced by contextual and cultural factors, which shape both learning processes and entrepreneurial outcomes ( Barba-Sánchez & Atienza-Sahuquillo, 2018; Cardella et al., 2024).

From a theoretical perspective, these results are consistent with the Theory of Planned Behavior, which highlights the role of individual perceptions, attitudes, and social norms in shaping entrepreneurial intention. In this study, cultural barriers and gender-related norms appear to function as contextual constraints that influence these cognitive and behavioral mechanisms.

Furthermore, the findings extend prior literature by emphasizing the role of cultural dynamics as a structuring dimension in entrepreneurial education. While previous studies have focused on psychological or institutional factors, this research integrates cultural barriers, perceptions, and transformation strategies into a unified framework, offering a more holistic understanding of entrepreneurial education processes.

Additionally, the results align with emerging research on gender and entrepreneurship, which suggests that deeply embedded social roles continue to influence women's entrepreneurial pathways ( Atienza-Barba et al., 2025). This highlights the need for educational models that incorporate gender-sensitive and culturally aware approaches to foster more inclusive entrepreneurial ecosystems.

The discussion is organised holistically in order to interpret the study’s findings in relation to the theoretical framework and the overall research objective. Firstly, an analysis of the results is presented, highlighting their relevance within the context of women’s entrepreneurial education. Subsequently, comparisons are made with national and international research, identifying both similarities and differences. In the following section, a conceptual framework is presented derived from the results obtained. This section also includes a discussion of the theoretical, political and practical implications. The limitations of the study are then described, and future research directions are proposed to further explore the topic.

### 4.1 Results analysis

The analysis of the results indicates that women’s leadership is characterised by a foundation in collaborative, transformational, and inclusive approaches that promote active participation and empowerment in educational and social contexts. This leadership programme has been shown to enhance critical thinking skills, encourage innovation, and foster self-management, all of which are vital to reducing the structural inequalities that persist in the entrepreneurial sphere. In accordance with extant research, entrepreneurial education has been demonstrated to promote gender equality and critical thinking in decision-making ( Medina-Vidal et al., 2023), in addition to fostering the development of innovative capacities in culturally diverse environments ( Li et al., 2021).

The analysis of the results indicates that sociocultural factors play a pivotal role in women’s entrepreneurial education, promoting empowerment, training, and social support as fundamental elements for enhancing women’s participation. The provision of entrepreneurial education has been demonstrated to engender heightened self-confidence and to foster gender equality through the facilitation of inclusive and collaborative learning environments. These results align with research demonstrating the positive influence of entrepreneurship education on entrepreneurial intent and the creation of initiatives with social impact ( Lan et al., 2023), as well as on the adoption of sustainable and equitable practices ( Makuya & Changalima, 2024).

The analysis of the results indicates that the primary constraints on women’s engagement in entrepreneurship education are rooted in institutional barriers, educational disparities, and cultural stereotypes that impede access and innovation. The findings of this study demonstrate that social structures and patriarchal norms perpetuate gender disparities in education and leadership. In accordance with recent studies, entrepreneurship education requires the implementation of diversified strategies and inclusive policies with the aim of overcoming structural biases and strengthening women’s self-efficacy ( Sun et al., 2025; Wang et al., 2023). Such measures are considered to be conducive to the promotion of equitable and sustainable educational environments.

The analysis of the results indicates that the provision of education in entrepreneurship for female members of the community has a significant impact on the community as a whole, with the effects of this being the strengthening of self-efficacy, economic empowerment, and collective resilience. The aforementioned effects are reflected in local growth, academic inclusion, and the establishment of social support networks. The findings align with research demonstrating that entrepreneurship education promotes economic and social well-being in vulnerable communities ( Tingey et al., 2020) and fosters the development of sustainable entrepreneurial intentions in students through university education and role models ( Amofah & Saladrigues, 2022), consolidating equitable and participatory ecosystems.

The analysis of the results indicates that programmes and approaches for the enhancement of entrepreneurship education for women prioritise the incorporation of a gender perspective, inclusive policies, and experiential learning. These strategies have been shown to encourage women’s participation, strengthen their self-confidence, and promote practical training in academic and business contexts. The findings align with research showing that entrepreneurial education increases entrepreneurial intention, especially among women and students from urban areas ( Deng & Wang, 2023), and strengthens self-efficacy as a determining factor in the decision to become entrepreneurs ( Ye & Kang, 2025), thus consolidating equitable and transformative educational environments.

### 4.2 Comparison of results with other studies

The present study corroborates earlier research by underscoring the impact of structural, symbolic, and community factors on the evolution of female leadership. Moreover, it extends the existing discourse by incorporating entrepreneurial education as a transformative component of social and economic advancement. In contrast to the findings of Wulandari and Ahmad (2025), who emphasised digital and technological access disparities, this analysis focuses on institutional and cultural impediments. In contrast to the focus of Aljohani and Alharbi (2025) on financial self-efficacy, this study emphasises collaborative learning and educational equity. Moreover, it builds upon the contributions of Djatmiko et al. (2025) on digital inclusion by underscoring the pivotal role of social support and community networks in women’s empowerment. In contrast to the analysis of technological innovation by Nazir et al. (2025), this study prioritises education and cultural transformation as cornerstones of sustainability. In accordance with the findings of Deng et al. (2025), it acknowledges the significance of perceived capabilities and gender equity, incorporating a comprehensive educational dimension that links self-efficacy with community leadership.

### 4.3 Proposed conceptual framework

**Figure 7.**
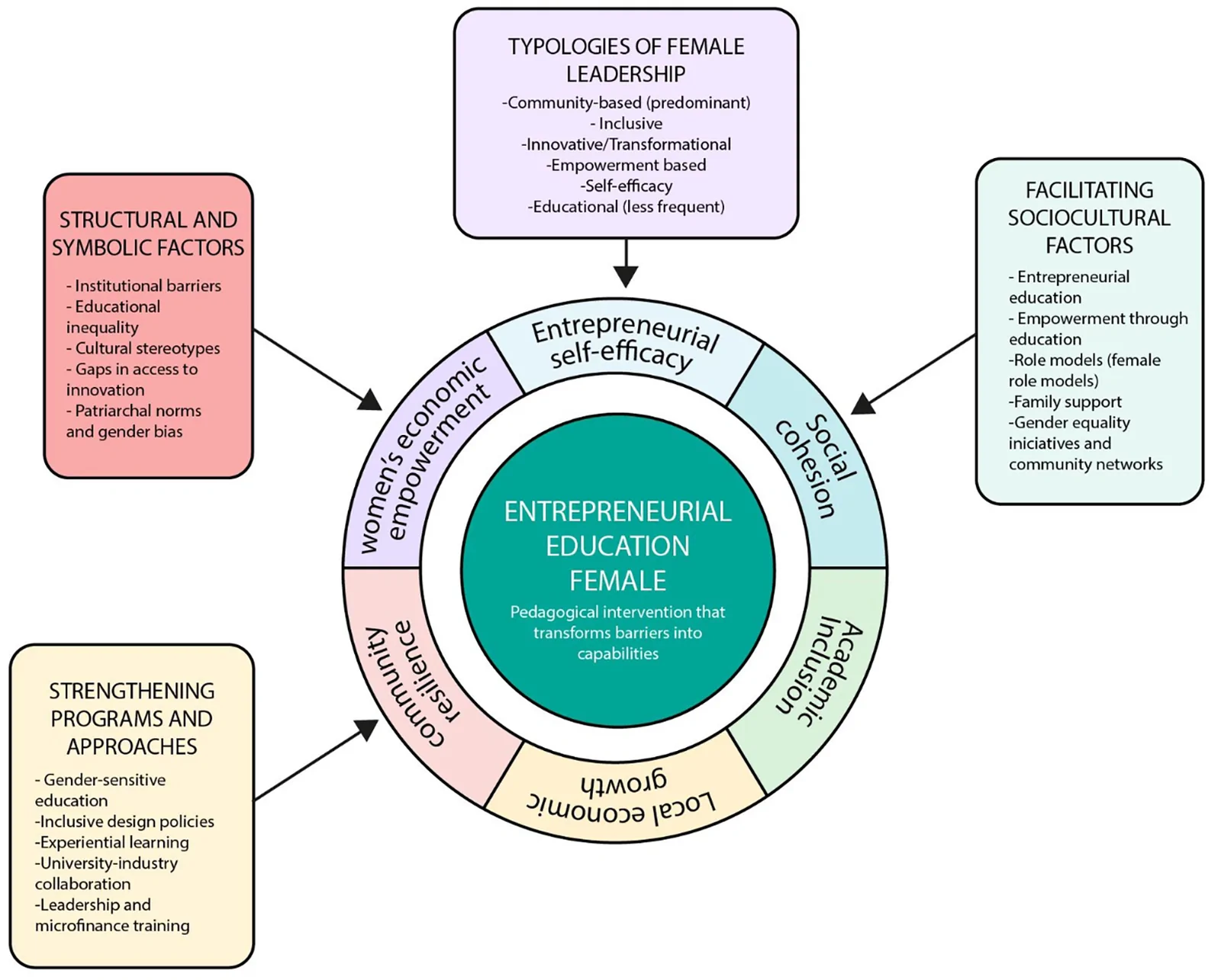
Conceptual framework of female entrepreneurial education. Prepared by the author based on the research results.

As illustrated in Figure 7, the proposed conceptual model integrates the determining factors of women’s entrepreneurial education. The framework under discussion articulates the structural, symbolic, sociocultural, and empowerment dimensions, reflecting their interaction in shaping women’s leadership. Entrepreneurial education is presented as a mediating force that transforms institutional and cultural barriers into opportunities for empowerment, equity, and sustainability, driving a continuous process of social transformation based on training, innovation, and community collaboration.

Building upon the systematic literature review, this study proposes a set of conceptual propositions that articulate the relationships between the key dimensions identified in female entrepreneurial education. These propositions are grounded in the qualitative synthesis of prior research and aim to provide a structured theoretical basis for the proposed conceptual framework. The conceptual framework is complemented by a set of propositions (P1–P5), which are visually represented in the model to illustrate the relationships between the identified dimensions.

• P1. Cultural barriers negatively influence women's participation and engagement in entrepreneurial education processes.
• P2. Individual perceptions, including self-efficacy, motivation, and perceived social norms, mediate the relationship between cultural context and entrepreneurial intention among women.
• P3. Entrepreneurial education programs that incorporate gender-sensitive and context-aware approaches positively influence women's entrepreneurial competencies and intentions.
• P4. Institutional and educational transformation strategies contribute to reducing cultural barriers and fostering inclusive entrepreneurial ecosystems.
• P5. The integration of cultural awareness within entrepreneurial education enhances the effectiveness of learning outcomes and supports women's entrepreneurial development.

### 4.4 Implications

#### 4.4.1 Theoretical Implications

This study contributes to the literature on entrepreneurial education by addressing critical gaps in the understanding of cultural, gender, and institutional barriers that shape women’s entrepreneurial intentions. Previous research has often focused on individual psychological factors or institutional constraints in isolation, but this study integrates these elements into a unified framework. The findings extend the Theory of Planned Behavior by emphasizing the role of cultural and gendered social norms, which mediate the influence of entrepreneurial education on women’s intentions. Furthermore, this research contributes to the growing body of literature on gender and entrepreneurship, highlighting how education can serve as a transformative force to challenge entrenched cultural biases and promote gender equality in entrepreneurial ecosystems.

#### 4.4.2 Methodological Implications

From a methodological perspective, this study introduces a novel approach to the analysis of entrepreneurial education by combining qualitative data synthesis with a conceptual framework that integrates cultural and gender-related factors. The use of structured thematic coding, despite not relying on specialized software, ensured transparency, replicability, and rigorous analysis. This study demonstrates that manual coding combined with systematic pivot table analysis is a valid methodological approach for mapping complex educational phenomena, and can be replicated in other contexts, particularly in studies involving gender-sensitive education. Additionally, the integration of cultural perspectives into the study of entrepreneurship education offers a valuable methodological tool for researchers interested in exploring the intersection of education, culture, and gender.

#### 4.4.3 Practical Implications

The practical implications of this study are significant for educational policymakers, institutions, and practitioners aiming to foster more inclusive entrepreneurial ecosystems. The findings suggest that women’s participation in entrepreneurial education can be enhanced through gender-sensitive curricula and inclusive policies that address both institutional and cultural barriers. This study also highlights the importance of integrating collaborative learning, experiential education, and social support networks into entrepreneurial programs. These insights provide actionable recommendations for universities, particularly in regions with significant gender disparities, to create educational environments that empower women and equip them with the skills and confidence necessary for entrepreneurial success. Furthermore, the findings underline the need for institutional reforms that promote gender equity and cultural transformation, offering valuable guidelines for stakeholders in both educational and entrepreneurial sectors.

### 4.5 Limitations and possible lines of future research

While this study makes significant contributions to the understanding of women’s entrepreneurial education, there are several limitations that warrant consideration. First, the study’s focus on university students limits the generalizability of the findings to broader populations, particularly those in early-stage entrepreneurial ventures or individuals with prior entrepreneurial experience. Future research could address this gap by including participants from various entrepreneurial stages, such as nascent entrepreneurs or women leading established businesses, to provide a more comprehensive view of the factors influencing entrepreneurial intentions.

Second, while this study examines cultural and gender barriers, it does not fully explore the intersectionality of these factors with other dimensions, such as socioeconomic status, ethnic background, or geographic location. Future studies could integrate these additional variables to examine how multiple social identities shape women’s engagement in entrepreneurial education and practice. The inclusion of diverse populations could help uncover more nuanced insights into the structural challenges faced by different groups of women in entrepreneurial contexts.

Third, although the study employs a robust methodological approach, it relies on self-reported data, which can be subject to biases such as social desirability or respondent interpretation. Future research could complement these findings with mixed-methods approaches, including qualitative interviews or ethnographic studies, to deepen the understanding of women’s lived experiences in entrepreneurial education. This would allow for more in-depth insights into how women navigate educational environments and overcome barriers in practice.

Finally, while the study identifies several strategies for enhancing women’s entrepreneurial education, it does not evaluate the effectiveness of these interventions in real-world settings. Future work could involve longitudinal studies or pilot programs to assess the impact of gender-sensitive and culturally inclusive entrepreneurial education programs on women’s entrepreneurial outcomes. By implementing and evaluating these interventions, researchers can gain a better understanding of how educational reforms can lead to lasting changes in women’s entrepreneurial participation and success.

## 5. Conclusions

The study’s findings demonstrate that entrepreneurial education for women constitutes a strategic space for social transformation, articulating efforts aimed at reducing gender inequalities, strengthening leadership, and promoting inclusive innovation. This process is not confined to the economic goals of entrepreneurship; rather, it establishes itself as a means of symbolic, educational, and community emancipation, linked to social development and collective well-being. In order to comprehend this dynamic, it is necessary to acknowledge that entrepreneurial capacities are developed in interaction with structural, cultural, and political factors. These factors determine the possibilities for training, participation, and leadership.

The analysis emphasises the necessity to redefine entrepreneurial training models with a focus on equity and social justice, thereby positioning women as transformative agents within educational and productive ecosystems. This change necessitates the interrogation of prevailing narratives that perpetuate women’s exclusion, and the promotion of collaborative networks among institutions, communities, and productive sectors that strengthen autonomy and resilience. The present paper sets out a definition of female entrepreneurship education as a collective process aimed at creating equitable, sustainable and inclusive environments where leadership is conceived as a tool for social innovation and cultural transformation.

## Ethical considerations

Ethical approval and Consent were not required.

## Data Availability

This study is based on source records retrieved from the Scopus and Web of Science databases, together with screening logs, data extraction tables, and PRISMA documentation generated throughout the review process. These materials support the identification, selection, and synthesis of the studies included in the analysis and ensure transparency in each methodological stage, from the initial search to the final thematic interpretation. The source records from Scopus and Web of Science are subject to database licensing and copyright restrictions and therefore cannot be considered fully open or reusable datasets under an open license. Although these records were cleaned, coded, and analyzed by the authors, their original source remains protected by the terms of use of the respective databases. For this reason, the full datasets cannot be redistributed freely but are shared exclusively for transparency and verification purposes. No individual, sensitive, or personal data were collected at any stage of this research, and the study did not involve human participants, interventions, or clinical data. Consequently, ethical approval or review by an Institutional Review Board (IRB) or equivalent ethics committee was not required. All procedures were conducted in accordance with international standards for research integrity and responsible data management. Aggregated screening counts, the PRISMA 2020 flow diagram, and the completed PRISMA 2020 checklist are publicly available in the Zenodo repository at https://doi.org/10.5281/zenodo.18625254 ( Valencia-Arias et al., 2025). Data are available under the terms of the Creative Commons Attribution 4.0 International license (CC-BY 4.0).
