# Cultural dynamics in entrepreneurial education: Barriers, perceptions, and transformation pathways

**DOI:** 10.12688/f1000research.178134.3

**Published:** 2026-08-03

**Authors:** Alejandro Valencia-Arias, Ada Gallegos, Jackeline Valencia, Ledy Gómez-Bayona, Rutsmy Angel Manuel Gallegos Pacheco

**Affiliations:** 1 Instituto de Investigación de Estudios de la Mujer, Universidad Ricardo Palma, Santiago de Surco, 15039, Peru; 2 Vicerrectoría de Investigación y postgrado, Universidad de Los Lagos, Osorno, Los Lagos Region, 5290000, Chile; 3 Coordinación de Investigación, Institucion Universitaria Marco Fidel Suarez, Medellín, Antioquia, 50010, Colombia; 4 Enfermería, Universidad Nacional de Educacion Enrique Guzman y Valle, Lima District, Lima Region, 15453, Peru

**Keywords:** Women's entrepreneurship education, cultural barriers, female leadership, gender equality, social transformation

## Abstract

Women’s entrepreneurial education has been increasingly recognized as a mechanism for promoting gender equality and economic participation in rural contexts. However, persistent cultural, symbolic, and structural barriers continue to limit women’s access to training, leadership opportunities, and innovation ecosystems, particularly in Latin America.

**Methods:**

A systematic review was conducted following the PRISMA 2020 guidelines. Searches were performed in Scopus and Web of Science between January and September 2025. Studies published between 2015 and 2025 in English or Spanish were included if they addressed women’s entrepreneurial education, leadership, or cultural barriers in rural or comparable contexts. A total of 842 records were identified, and after screening and eligibility assessment, 129 studies were included in the qualitative synthesis and 45 in the in-depth analysis.

**Results:**

The findings indicate that institutional constraints, educational inequalities, and persistent gender stereotypes are the most frequently reported barriers. Facilitating factors include sociocultural support, visible role models, inclusive educational programs, and gender-oriented public policies. Women’s leadership is commonly described as collaborative and community-oriented, with documented associations to entrepreneurial self-efficacy, economic participation, and community resilience.

**Conclusions:**

Entrepreneurial education functions as a mediating mechanism within rural ecosystems, transforming structural and symbolic barriers into opportunities for empowerment and leadership. These findings contribute to a more comprehensive understanding of the cultural and institutional conditions shaping women’s entrepreneurial education and leadership in rural contexts.

## 1. Introduction

Women’s entrepreneurial education has been identified as a fundamental component of sustainable development, promoting economic autonomy, leadership, and women's active participation in the social and productive transformation of communities. In rural contexts, the scope of this educational approach extends beyond the mere establishment of business enterprises. It encompasses the acquisition of competencies, principles, and mindsets that fortify the capacity to generate innovative solutions, adapt to environmental change, and spearhead local development initiatives. Consequently, it is configured as a strategic axis for gender equality, contributing to the closure of gaps in access to education, employment, and productive resources ( Miran & Gültekin, 2024; Sheena, n.d.). Nevertheless, the consolidation of women’s entrepreneurial education is confronted by numerous cultural barriers that impede women’s participation and leadership. These include gender stereotypes, patriarchal structures, the sexual division of labour, and the low value placed on women’s knowledge in rural environments.

These conditions have been shown to restrict the development of entrepreneurial skills and diminish the visibility of women as agents of change ( Christodoulou et al., 2024; Nevi et al., 2025). This is consistent with research demonstrating the persistence of social and cultural biases that affect entrepreneurial motivations, opportunities, and strategies across contexts. The analysis of this phenomenon from a rural perspective facilitates understanding of how cultural dynamics influence the sustainability of productive projects and the social transformation of communities. Within this framework, entrepreneurial education is aligned with the Sustainable Development Goals, particularly SDG 5 (Gender Equality) and SDG 8 (Decent Work and Economic Growth), as well as with national policies aimed at inclusion, empowerment, and territorial equity. This approach underscores the need to implement educational and leadership strategies that address sociocultural barriers, empower women’s innovation, and fortify rural entrepreneurial ecosystems ( Christodoulou et al., 2024; Nevi et al., 2025).

The importance of entrepreneurial education for women in achieving sustainable development and reducing inequalities is widely acknowledged. However, a knowledge gap persists regarding the limited integration of cultural and leadership dimensions in rural contexts. The extant literature addresses the role of women in entrepreneurial processes in a fragmented manner, failing to sufficiently integrate the sociocultural factors that influence their participation and the dynamics of community leadership. This disconnection creates gaps in understanding cultural structures, belief systems, and social mores that influence the consolidation of projects led by rural women, thereby curtailing the formulation of policies and programs tailored to their realities ( Naguib, 2024; Abd El Basset et al., 2024). Empirical evidence demonstrates that the underrepresentation of women in rural entrepreneurship is associated with cultural and symbolic factors that perpetuate unequal power relations.

Patriarchal structures continue to exert a significant influence on the social and economic roles of men and women, thereby restricting women’s autonomy and decision-making power. Furthermore, the sexual division of labour and the undervaluation of knowledge generated by women serve to reinforce their exclusion from economic and political leadership positions. In many cases, the phenomenon of women’s entrepreneurship is perceived as an extension of domestic work or a secondary activity, thereby reinforcing the idea of a subordinate role in local economic development ( Miralam et al., 2025; Ahmed et al., 2025).

Moreover, a paucity of integration has been identified among educational, social, and economic approaches aimed at enhancing women’s participation in rural areas. It is evident that entrepreneurship education programmes tend to prioritise the cultivation of technical and financial competencies. However, there is a conspicuous absence of emphasis on the symbolic barriers, socialisation patterns, and power structures that impede knowledge acquisition. This absence of coherence limits the creation of transformative processes that incorporate women’s leadership as a strategic component of social change ( Naguib, 2024; Miralam et al., 2025).

To comprehend the cultural patterns that influence women’s entrepreneurial education and the configuration of rural leadership, a comparative and contextual analysis is necessary. This analysis facilitates the identification of mechanisms of exclusion and the strategies of cultural resistance developed by women, thereby providing theoretical and empirical evidence for the re-definition of educational and community practices. The knowledge gap concerns the limited understanding of the interactions among education, culture, and rural women’s leadership, underscoring the relevance of a study that examines these links from a transformative perspective ( Abd El Basset et al., 2024; Ahmed et al., 2025).

Entrepreneurial education has become a critical mechanism for fostering economic growth, innovation, and job creation, particularly in contexts characterised by uncertainty and structural transformations. Previous studies have highlighted that entrepreneurship contributes not only to economic development but also to social value creation and improved quality of life ( Cardella et al., 2024). In this sense, universities play a key role in promoting entrepreneurial competencies and intentions among students, positioning entrepreneurial education as a strategic priority in higher education systems.

Entrepreneurship education has become a key mechanism for strengthening economic participation, innovation, employability, and the creation of social value, particularly in contexts marked by uncertainty, inequality, and structural transformation. Its relevance lies not only in promoting business creation but also in developing the motivation, competencies, attitudes, and decision-making skills that enable people to identify opportunities and respond to changing social and economic conditions. In this regard, Barba-Sánchez & Atienza-Sahuquillo (2018) show that entrepreneurship education positively contributes to entrepreneurial intention by strengthening motivational and competency-based factors, confirming the strategic role of educational institutions in preparing students for entrepreneurial careers.

Recent evidence also suggests that entrepreneurial education should be understood in relation to contextual, psychological, institutional, and social factors. Cardella et al. (2024) demonstrate that adverse environments, such as the COVID-19 pandemic, can negatively affect entrepreneurial intention, whereas psychological resources, such as optimism and proactivity, can help students cope with uncertainty and maintain entrepreneurial motivation. Likewise, Atienza-Barba et al. (2025) highlight that environmental awareness and gender differences influence entrepreneurial intention, suggesting that entrepreneurship education should integrate sustainable, inclusive, and gender-sensitive approaches.

Furthermore, recent reviews have advocated broader, more integrative approaches to entrepreneurship education. Passarelli and Bongiorno (2025) argue that the existing literature has often focused on isolated factors at the micro or macro level, whereas the field increasingly requires multidisciplinary frameworks capable of addressing new social, environmental, and market challenges. Similarly, Serpente et al. (2025) highlight that entrepreneurship education and training also take place outside traditional classrooms, particularly through entrepreneurship support organisations, where theoretical knowledge, practical learning, mentoring, and ecosystem-based support are brought together.

In this regard, these contributions are particularly relevant to the present study, as women’s entrepreneurship education in rural contexts is shaped not only by individual motivation, institutional support, and pedagogical design, but also by cultural norms, symbolic barriers, gender roles, and leadership opportunities. Therefore, the literature shows that examining cultural barriers, women’s perceptions, and pathways to transformation is essential to understanding how entrepreneurship education can serve as a mechanism for empowerment, inclusion, and social transformation.

Despite these advances, there is still a need for integrative approaches that place cultural dynamics at the centre of entrepreneurship education. Previous studies have primarily examined entrepreneurial intention, psychological resources, sustainability, institutional support, or educational ecosystems as isolated dimensions. However, less is known about how cultural barriers, women’s perceptions, leadership dynamics, and transformation strategies interact in rural and similar contexts, where gender roles, symbolic norms, and institutional inequalities can significantly influence participation, learning, and entrepreneurial engagement.

To address this gap, this study offers an integrative systematic review that connects cultural barriers, perceptions, and pathways to transformation within a unified analytical framework. Its contribution lies in going beyond the identification of isolated constraints to propose a multidimensional perspective on women’s entrepreneurship education. In this way, the study provides a foundation for understanding how culturally sensitive and gender-responsive educational strategies can support empowerment, inclusive leadership, and social transformation in rural contexts.

With all of this in mind, although research on women’s entrepreneurship education has expanded considerably, significant gaps remain. First, most studies have examined entrepreneurial intention, self-efficacy, institutional support, and gender differences as isolated dimensions, while cultural and symbolic barriers are often treated as contextual rather than central analytical categories. Second, previous reviews and empirical studies have focused on the effectiveness of entrepreneurship education or on women’s entrepreneurship in general, without sufficiently linking cultural barriers, women’s perceptions, leadership models, and transformation strategies within a unified framework. Third, evidence on rural and similar contexts—particularly in Latin America and other regions marked by structural inequalities—remains scattered, which limits the design of educational interventions and policies tailored to these contexts.

This study differs from previous research by offering an integrative systematic review that connects three dimensions that are generally analysed separately: cultural barriers, perceptions of women’s entrepreneurship education, and pathways of transformation toward inclusive leadership. Its main contribution is the development of a multidimensional conceptual framework that explains the interaction between structural, symbolic, institutional, and sociocultural factors in women’s entrepreneurship education. In this regard, the study goes beyond identifying obstacles and provides a synthesised foundation for the design of gender-sensitive, culturally responsive, and context-specific entrepreneurship education programs.

The objective of this research is to identify the cultural barriers and transformative factors that influence entrepreneurial education and women’s leadership in rural Latin American contexts. In order to achieve this objective, a series of questions has been devised to guide understanding of the cultural barriers and transformative factors associated with women’s leadership in rural contexts.

1. What characteristics define the most frequent leadership models among rural women according to the academic literature?
2. What social, cultural, or institutional factors have facilitated the emergence of women’s leadership in rural areas?
3. What barriers have limited the recognition or effective participation of rural women in community or political leadership spaces?
4. What impacts have been documented in communities where rural women hold active leadership roles?
5. What approaches or programs have been implemented to strengthen leadership capacities in rural women?

## 2. Methodology

The research employed the PRISMA 2020 (Preferred Reporting Items for Systematic Reviews and Meta-Analyses) methodology as a guide for the systematic review, in order to ensure transparency, rigour, and reproducibility in the search, selection, and synthesis of scientific evidence. This methodological approach provides a structured framework that facilitates the identification, evaluation, and coherent presentation of results obtained from verified academic sources, guaranteeing the traceability of the entire process. The application of the PRISMA model enabled the analysis of studies on cultural barriers, entrepreneurial education, and female leadership in rural Latin American contexts, following the four phases outlined in the protocol: identification, screening, eligibility, and inclusion. These phases enabled the refinement of the information and the final selection of the most relevant articles ( Page et al., 2021).

### 2.1 Eligibility criteria

The selection of studies for the review was based on eligibility criteria designed to ensure relevance, quality, and coherence with the research objectives. The inclusion criteria encompassed peer-reviewed articles published between 2015 and 2025, written in English or Spanish, that addressed entrepreneurial education, women’s entrepreneurship, leadership, and cultural barriers. The study incorporated a range of research methodologies, including qualitative, quantitative, and systematic review approaches, with the overarching objective of presenting empirical or theoretical evidence on women’s participation in entrepreneurial training, rural leadership, or social transformation. The selection prioritised studies analysing the relationships among education, culture, and gender in local development contexts, with a particular focus on Latin America, Africa, and Asia, to identify global patterns and regional specificities. The parameters for the literature search and evaluation were established to ensure the validity and traceability of the results.

It is important to note that only studies from academic databases recognised for their rigour and scientific visibility were included. This ensured the reliability of the evidence used. The selected documents addressed the phenomenon from social, cultural, and institutional perspectives, supported by consistent theoretical frameworks and verifiable methodologies. A range of studies exploring the intersection of gender, education and economic development were also considered, with a particular emphasis on how cultural factors influence leadership and entrepreneurship opportunities for rural women. The exclusion process was executed in three successive phases.

In the initial phase of the study, articles containing indexing errors or duplicate records were eliminated. In the second instance, those without full-text access were excluded because their methodological content could not be analysed. In the third study, an exclusion criterion was applied. This criterion was based on thematic relevance, theoretical depth, and the absence of novel contributions compared to other studies. The implementation of these methodologies resulted in a comprehensive, high-quality set of studies, thereby ensuring a robust methodological foundation for analysing cultural barriers and transformative factors in women’s entrepreneurial education across diverse global regions.

### 2.2 Sources of information

The systematic review was based on two highly visible and internationally recognised academic databases: Scopus and Web of Science. The selection of these platforms was based on their thematic and geographic coverage, as well as their relevance to scientific production in the areas of social sciences, education, and business studies. It is important to note that both databases are regarded as benchmarks for evaluating scientific knowledge. In addition, they provide access to publications that have been peer-reviewed. This ensures the reliability, traceability and consistency of the retrieved information. The amalgamation of these two sources enhanced the representativeness of the included studies, thereby mitigating biases that might have arisen from editorial bias. This approach ensured a more comprehensive coverage of regions and disciplines ( Asubiaro, Onaolapo, & Mills, 2024). The search was conducted between January and September 2025, incorporating original articles, systematic reviews, and academic chapters that contributed empirical or theoretical evidence on women’s entrepreneurial education, rural leadership, and cultural barriers.

The process entailed a comprehensive review of the available records in each database, with pre-established inclusion and exclusion criteria being applied to ensure consistency and methodological rigour. Scopus and Web of Science both offer comprehensive metadata, including authorship, year of publication, country, institutional affiliation, subject area, number of citations, and document type. These elements enabled the organisation and classification of results according to scientific and geographical variables. This configuration enabled the refinement of records and the identification of emerging trends in literature. Publications in journals ranked in the Q1 and Q2 quartiles of the Scimago Journal Rank (SJR) were prioritised, thereby ensuring the selection of high-impact studies demonstrating methodological quality. The search was complemented by a manual search in Google Scholar, aimed at locating grey literature and recent works on rural women’s leadership and cultural transformation.

### 2.3 Search strategy

A specific search formula was established for each database, formulated in accordance with the inclusion criteria and the PRISMA 2020 guidelines. The search strategy was designed to capture the core focus of the review: entrepreneurship education and women’s participation. Consequently, the main search of the databases focused on terms directly related to entrepreneurship education and gender. In Scopus, the search formula used was TITLE (“entrepreneurial education” OR “entrepreneurship education”) AND TITLE-ABS-KEY (“women” OR “female”), whilst in Web of Science, the equivalent structure was TS=(“entrepreneurial education” OR “entrepreneurship education”) AND TS=(“women” OR “female”). This strategy was chosen to ensure thematic accuracy and to avoid retrieving an excessively broad corpus of studies on entrepreneurship, leadership, rural development or empowerment that were not directly related to entrepreneurship education.

Whilst concepts such as culture, leadership, empowerment, rural development and social transformation were not treated as separate search blocks in the main database queries, they were incorporated through the eligibility criteria, full-text review, supplementary manual searches and thematic coding. Specifically, studies were retained that addressed women’s entrepreneurship education in relation to cultural barriers, gender stereotypes, leadership dynamics, empowerment processes, rural or community development, institutional constraints or social transformation. Supplementary manual searches and reference checking were also used to identify relevant studies linking entrepreneurship education to these broader sociocultural dimensions.

This approach enabled the review to maintain a focused search strategy, whilst incorporating the broader analytical dimensions necessary to answer the research questions. In this way, culture, leadership and empowerment were operationalised not only as search-related concepts, but also as eligibility and coding categories used to refine the final corpus and guide the qualitative synthesis. This procedure strengthened the coherence between the search protocol, the selection process and the thematic structure of the findings.

### 2.4 Selection process

**Figure 1.**
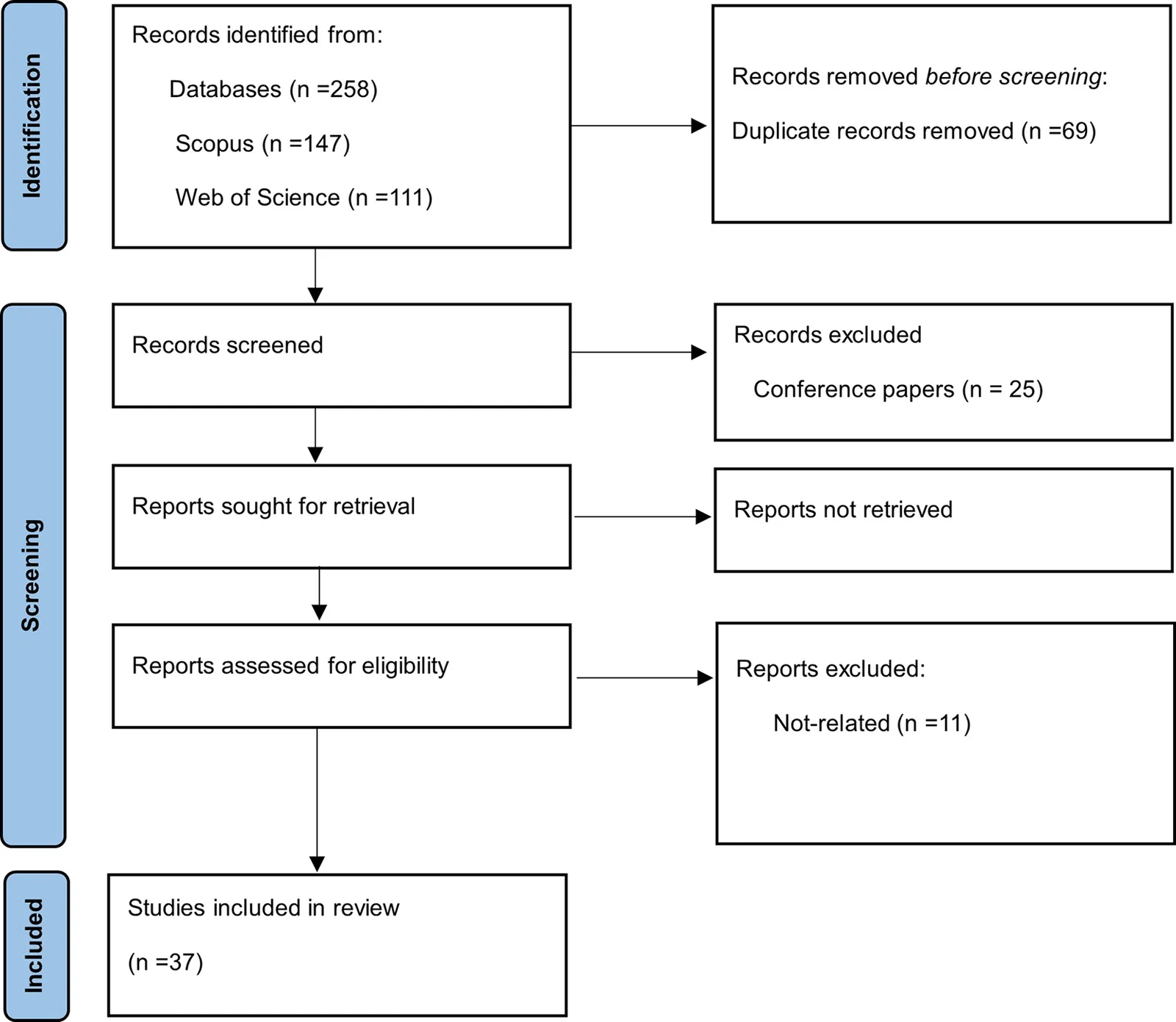
PRISMA flowchart. Created by the author using data from Scopus and Web of Science.

The selection of studies was carried out in three consecutive stages, following a systematic procedure designed to guarantee the relevance and quality of the final corpus. In the initial phase, the titles and abstracts were reviewed to ensure thematic consistency and remove redundancies across the various databases. In the subsequent stage, the complete texts of the selected articles from the first stage were analysed. The methodological consistency and coherence of these articles with the defined inclusion criteria were assessed. In the third stage, the final selection was made based on the theoretical relevance and scientific rigour of each document. The review process was overseen by two researchers, who conducted independent reviews of each record to minimise bias. Disagreements were resolved through consensus. The entire procedure is shown in Figure 1, which corresponds to the PRISMA 2020 flowchart.

A total of 842 records were identified from the Scopus (472) and Web of Science (370) databases. A further 18 records were incorporated into the study through a manual search of Google Scholar and a review of bibliographic references. During the preliminary data cleansing stage, 112 duplicate records were removed, leaving 748 unique studies for review. In the screening phase, titles and abstracts were examined, and 421 articles were excluded for lack of relevance to women’s entrepreneurial education, leadership, or cultural barriers. In the eligibility phase, 327 full texts were analysed, and 198 were excluded for not meeting the inclusion criteria, either due to indexing errors, restricted access, or a lack of focus on rural women’s leadership. In the final analysis, 129 studies were included in the qualitative synthesis, and 45 articles were included in the detailed analysis of results. The complete process is shown in Figure 1, which corresponds to the PRISMA 2020 flow diagram.

### 2.5 Quality appraisal of the included studies

To enhance the transparency and rigour of the review process, the quality of the included studies was assessed. Given the methodological diversity of the body of research, which included quantitative, qualitative, mixed-methods, conceptual, review-based and policy-oriented studies, a tailored quality assessment checklist was developed, based on principles widely used to assess methodological clarity, consistency and transparency in evidence synthesis.

Each study was assessed according to five criteria: (1) clarity of the research objective; (2) appropriateness of the methodological or conceptual design; (3) suitability of the data collection process, sample, corpus or information sources; (4) consistency between the analytical procedure and the findings or conclusions; and (5) transparency in the presentation of limitations and conclusions. Each criterion was scored as 1 for full compliance, 0.5 for partial compliance, and 0 for noncompliance or insufficient reporting. The maximum possible score was 5 points (see Table 1).

**Table 1.** Methodological assessment of the included articles. Prepared by the author using data from Scopus and Web of Science.

Title	Approach	Objective clear	Design appropriate	Data/source adequate	Analysis coherent	Reporting transparent	Score	Reliability level
Analyzing the role of gender in entrepreneurship education and economic success in developing nations: the case of Colombia	Quantitative empirical study	Yes	Yes	Partial	Partial	Yes	4.0/5	High
Creating Shared Value in Banking by Offering Entrepreneurship Education to Female Entrepreneurs	Qualitative case study	Yes	Yes	Yes	Yes	Partial	4.5/5	High
Does entrepreneurship education in China develop entrepreneurial intention? The role of self-efficacy and experience	Quantitative empirical study	Yes	Yes	Yes	Yes	Yes	5.0/5	High
Entrepreneurship education and entrepreneurial intention: Do female students benefit?	Quantitative empirical study	Yes	Partial	Yes	Yes	Yes	4.5/5	High
Entrepreneurship education in TVET institutions and entrepreneurial intentions of female students in Ghana: The social support factor	Mixed-methods cross-sectional study	Yes	Yes	Yes	Partial	Partial	4.0/5	High
Evaluating the Role of Entrepreneurship Education in Shaping Female College Students’ Career Choices in China: A Multidimensional Analysis	Quantitative empirical study	Yes	Yes	Partial	Partial	Partial	3.5/5	Medium
Financial Literacy as a Key to Entrepreneurship Education: A Multi-Case Study Exploring Diversity and Inclusion	Mixed-methods multi-case study	Yes	Yes	Partial	Yes	Partial	4.0/5	High
Gender Roles in Entrepreneurship Education to Social Entrepreneurial Intentions in Vietnam	Quantitative empirical study	Yes	Yes	Partial	Yes	Partial	4.0/5	High
Gendered Entrepreneurship Education and the Fear of Failure	Quantitative empirical study using secondary GEM data	Yes	Yes	Yes	Partial	Partial	4.0/5	High
Impact of Innovation and Entrepreneurship Education in a University Under Personality Psychology Education Concept on Talent Training and Cultural Diversity of New Entrepreneurs	Quantitative descriptive survey study	Yes	Partial	Partial	Partial	Yes	3.5/5	Medium
Improving the Entrepreneurial Competence of College Social Entrepreneurs: Digital Government Building, Entrepreneurship Education, and Entrepreneurial Cognition	Quantitative empirical study	Yes	Yes	Yes	Yes	Partial	4.5/5	High
Overcoming Gender Gaps in Entrepreneurship Education and Training	Qualitative study with primary and secondary data	Yes	Yes	Partial	Yes	Yes	4.5/5	High
The effect of entrepreneurship education on the entrepreneurial intention of different college students: Gender, household registration, school type, and poverty status	Quantitative empirical study	Yes	Yes	Partial	Yes	Yes	4.5/5	High
The Impact of Entrepreneurial Self-Efficacy and Entrepreneurship on Entrepreneurial Intention: Entrepreneurial Attitude as a Mediator and Entrepreneurship Education Having a Moderate Effect	Quantitative empirical study using SEM	Yes	Yes	Partial	Yes	Partial	4.0/5	High
Unveiling the Role of Entrepreneurship Education on Green Entrepreneurial Intentions among Business Students: Gender as a Moderator	Quantitative empirical study using PLS-SEM	Yes	Yes	Partial	Yes	Yes	4.5/5	High
College students’ entrepreneurship education path and management strategy of start-up enterprises using causal attribution theory	Quantitative analytical study using questionnaire, causal attribution theory, DL and AI	Yes	Partial	Partial	Partial	Partial	3.0/5	Medium
Educators and students in entrepreneurship education are challenging the “think entrepreneur–think male” paradigm	Qualitative single case study	Yes	Yes	Yes	Yes	Partial	4.5/5	High
Entrepreneurial, Economic, and Social Well-Being Outcomes from an RCT of a Youth Entrepreneurship Education Intervention among Native American Adolescents	Mixed-methods randomized controlled trial	Yes	Yes	Yes	Yes	Partial	4.5/5	High
Entrepreneurship Education among University Students as a Predictor of Female Entrepreneurial Undertakings	Conceptual literature-based study	Yes	Partial	Partial	Partial	Partial	3.0/5	Medium
Entrepreneurship Education and Disability: An Experience at a Spanish University	Quantitative empirical study	Yes	Partial	Partial	Yes	Yes	4.0/5	High
Entrepreneurship Education for Women—European Policy Examples of Neoliberal Feminism?	Qualitative policy analysis	Yes	Yes	Yes	Yes	No/Partial	4.0/5	High
Exploration and Practice of Maker Education Mode in Innovation and Entrepreneurship Education	Quantitative empirical study using questionnaire survey	Yes	Partial	Partial	Partial	Yes	3.5/5	Medium
Gender-sensitive vocational and entrepreneurship education: addressing poverty for Caribbean women	Exploratory integrative literature review	Yes	Partial	Partial	Yes	Yes	4.0/5	High
Generative artificial intelligence in entrepreneurship education enhances entrepreneurial intention through self-efficacy and university support	Quantitative empirical study using PLS-SEM	Yes	Yes	Partial	Yes	Yes	4.5/5	High
How does entrepreneurial curiosity stimulate new venture ideas among Chinese undergraduates? The mediating role of promotion focus and the moderating role of entrepreneurial education	Quantitative empirical study using PLS-SEM	Yes	Yes	Partial	Yes	Yes	4.5/5	High
Impact of attitude towards entrepreneurship education and role models on entrepreneurial intention	Quantitative empirical study using PLS-SEM	Yes	Yes	Partial	Yes	Partial	4.0/5	High
Impact of Personality Traits and Entrepreneurship Education on Entrepreneurial Intentions of Business and Engineering Students	Quantitative empirical study using logistic regression	Yes	Yes	Partial	Yes	Partial	4.0/5	High
Role Modeling as a Pedagogical Strategy in Entrepreneurship Education for Women and Girls	Conceptual pedagogical model	Yes	Partial	Partial	Partial	Partial	3.0/5	Medium
Teacher-student empathic relationship shaping: an elimination mechanism for psychological segmentations in entrepreneurship education	Quantitative experimental study	Yes	Yes	Partial	Yes	Partial	4.0/5	High
Teaching Through Television: Experimental Evidence on Entrepreneurship Education in Tanzania	Randomized controlled field experiment	Yes	Yes	Yes	Yes	Partial	4.5/5	High
The Analysis of the Effect of Entrepreneurship Education, Perceived Desirability, and Entrepreneurial Self-Efficacy on University Students’ Entrepreneurial Intention	Quantitative inferential cross-sectional study	Yes	Partial	Partial	Yes	Yes	4.0/5	High
The Development of Inclusive Agriculture Entrepreneurship Education Ecosystems for Young Entrepreneurs in Uganda	Qualitative interpretivist study using discourse analysis	Yes	Yes	Partial	Yes	Partial	4.0/5	High
The Effect of Entrepreneurship Education on Entrepreneurial Intention: Mediation of Entrepreneurial Self-Efficacy and Moderating Model of Psychological Capital	Quantitative cross-sectional study using SEM	Yes	Yes	Partial	Yes	Yes	4.5/5	High
The Influence of Economic and Entrepreneurial Education on Perception and Attitudes towards Entrepreneurship	Quantitative cross-sectional survey study	Yes	Yes	Partial	Yes	Partial	4.0/5	High
The Re-integration of College Innovation and Entrepreneurship Education Under Young Entrepreneurs’ Enterprising Spirit and Professional Music Education	Quantitative descriptive survey study	Yes	Partial	Partial	Yes	Partial	3.5/5	Medium
The Role of Entrepreneurship Education as a Predictor of University Students’ Entrepreneurial Intention	Quantitative empirical study using probit regression	Yes	Yes	Partial	Yes	Yes	4.5/5	High
Unpacking Entrepreneurial Education: Learning Activities, Students’ Gender, and Attitude Toward Entrepreneurship	Quantitative empirical study	Yes	Yes	Partial	Partial	Partial	3.5/5	Medium

Based on the total score, studies were classified into three levels of reliability: high reliability (4.0-5.0), medium reliability (3.0-3.5), and low reliability (<3.0). The assessment was not used as an exclusion criterion, but rather as a tool to evaluate the strength of the evidence and to distinguish between primary empirical evidence and complementary conceptual or contextual support. Discrepancies in classification were discussed amongst the researchers and resolved by consensus.

For conceptual, pedagogical or policy studies, the assessment focused on the clarity of the argument, the relevance of the sources used, the consistency between the literature reviewed and the proposed framework, and the transparency regarding limitations. This flexible procedure allowed the assessment to be comparable across different types of documents, whilst respecting their methodological specificities.

• Clarity of research objective: The study clearly states its objective, question or purpose.
• Appropriateness of design: The methodological, conceptual or analytical design is suitable for the objective.
• Adequacy of data or sources: The sample, corpus, documents or sources are sufficiently described and relevant.
• Coherence between analysis and findings: The findings or conclusions are logically derived from the analysis.
• Transparency in limitations and conclusions: The study reports limitations or clearly delimits the scope of its conclusions.

### 2.6 Data processing

Microsoft Excel was utilised as the primary tool for systematically documenting, organising, and structuring the data obtained during the review process. This approach ensured the implementation of a structured procedure that facilitated the traceability of the analytical process. The data were coded using precise variables, including author, year of publication, country, type of study, methodological approach, main results, and identified barriers, which enabled consistent classification and control of the consulted sources. The information was organised into pivot tables that facilitated comparative analysis and the generation of descriptive graphs to visualise trends. The findings were grouped into four thematic areas, namely cultural barriers, transformation strategies, entrepreneurial education, and women’s leadership, which facilitated the identification of recurring patterns, knowledge gaps, and emerging lines of research related to women’s participation in entrepreneurship and rural leadership processes.

Coding and thematic synthesis followed a combined deductive and inductive procedure. Firstly, an initial coding matrix was developed based on the research questions and the main analytical dimensions of the review: leadership models, socio-cultural enabling factors, structural and symbolic barriers, community impacts, and capacity-building programmes. Secondly, the full texts of the selected studies were reviewed to identify recurring concepts, patterns and findings relating to women’s entrepreneurship education. During this stage, additional sub-themes emerged inductively from the evidence, including role models, self-efficacy, institutional constraints, gender stereotypes, empowerment, community resilience and inclusive educational strategies.

To improve consistency, the coding categories were reviewed and refined iteratively throughout the analysis. The coding process was validated by two researchers. Disagreements regarding the classification of studies or the assignment of themes were discussed and resolved by consensus. This procedure ensured consistency between the research questions, the thematic categories and the interpretation of the findings.

**Figure 2.**
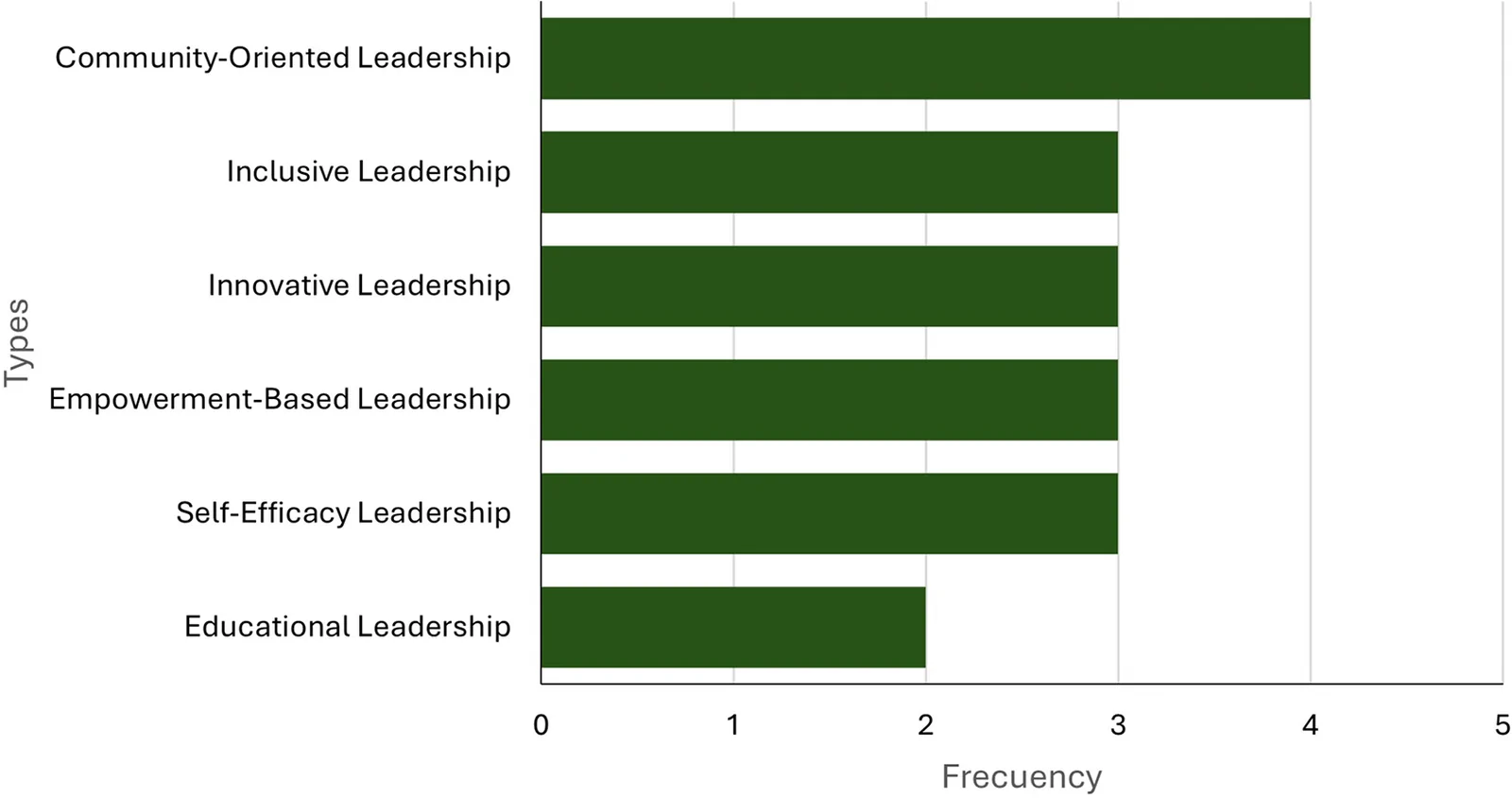
Distribution of female leadership typologies. Prepared by the author using data from Scopus and Web of Science.

**Figure 3.**
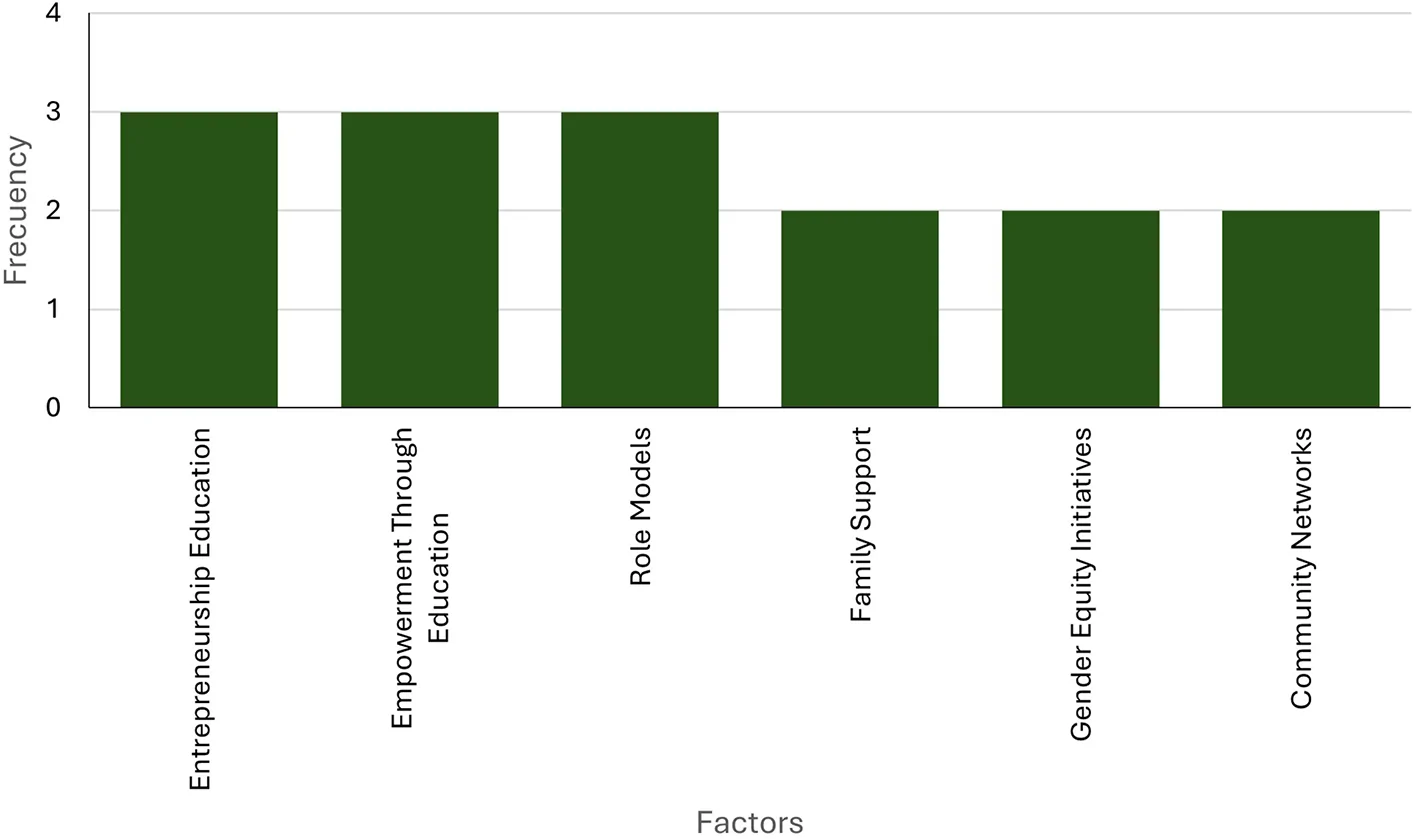
Sociocultural factors facilitating female entrepreneurship. Prepared by the author based on Scopus and Web of Science.

**Figure 4.**
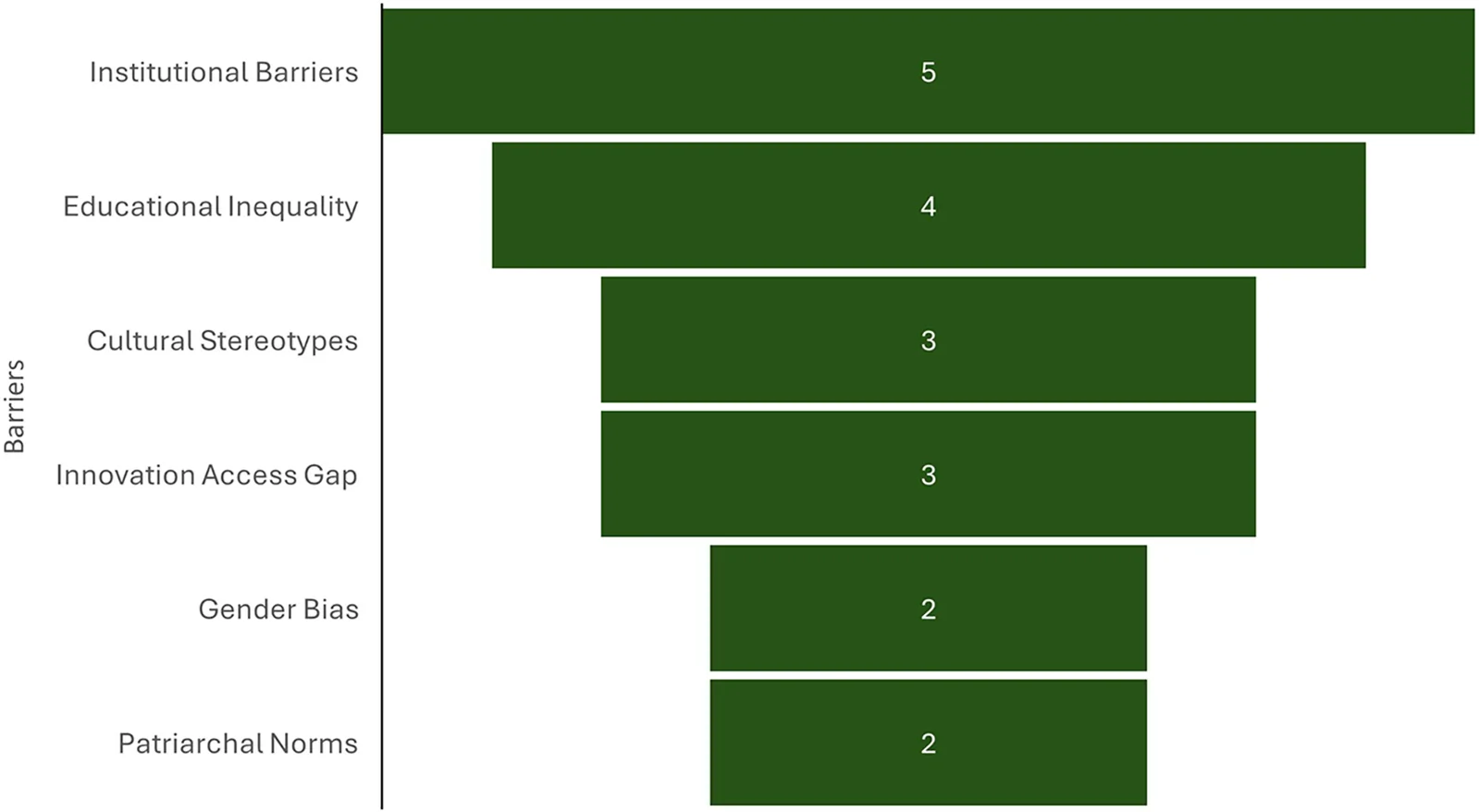
Structural and symbolic barriers in female entrepreneurial education. Prepared by the author based on Scopus and Web of Science.

**Figure 5.**
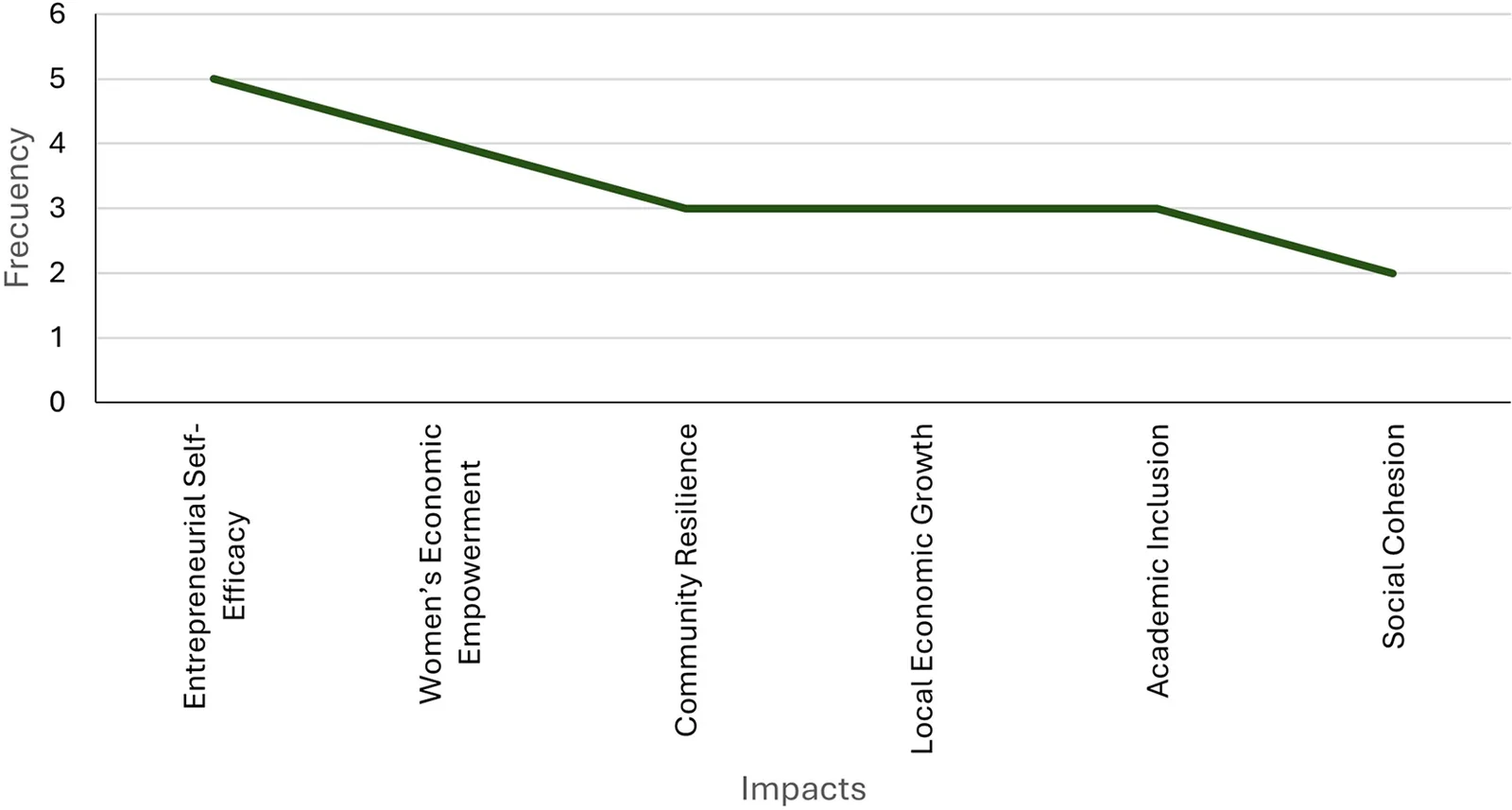
Documented community impacts on female entrepreneurial education. Prepared by the author based on Scopus and Web of Science.

**Figure 6.**
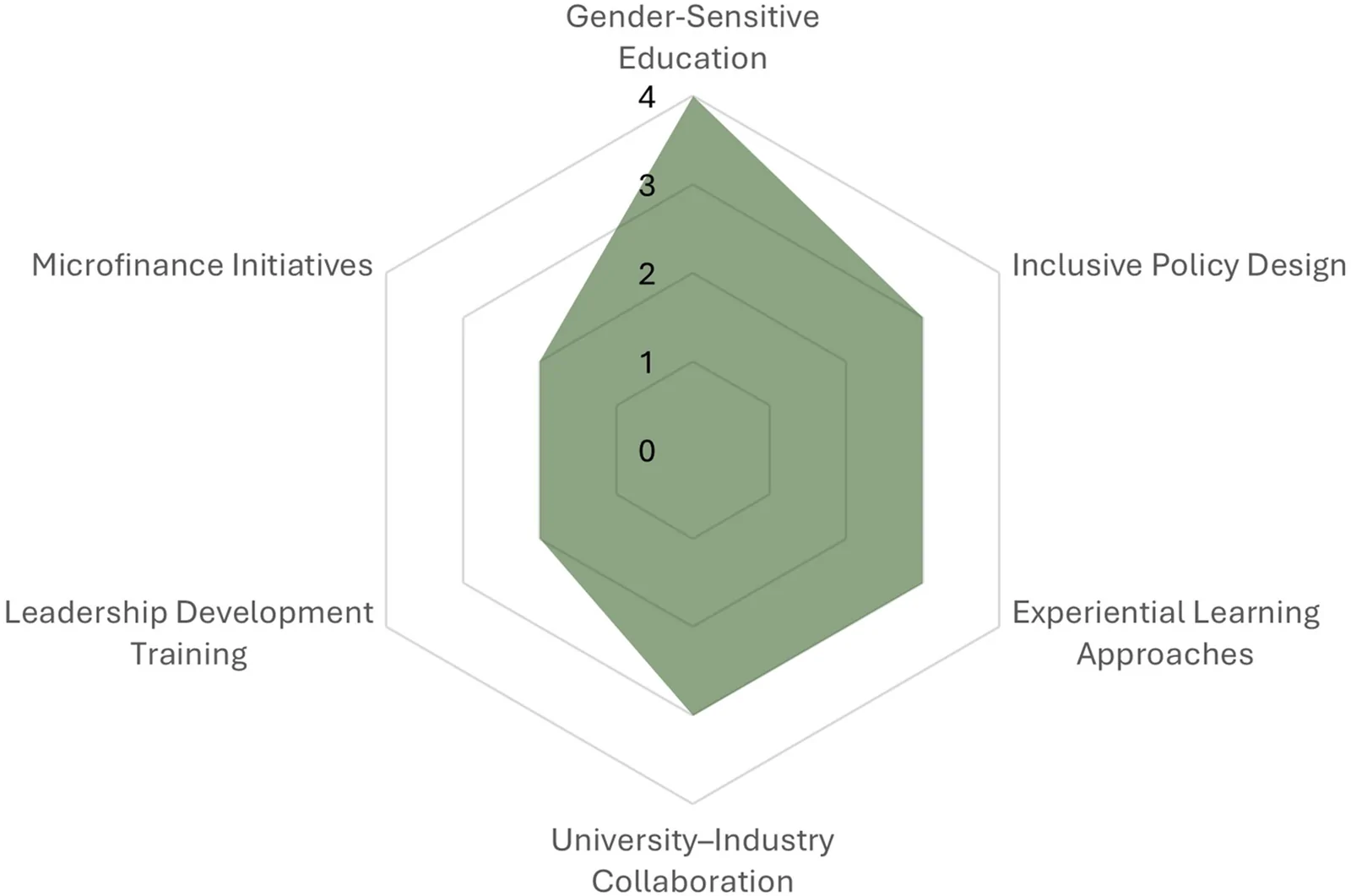
Programs and approaches for strengthening women's entrepreneurial education. Prepared by the author based on Scopus and Web of Science.

The frequencies presented in Figures 2 to 6 were calculated using the study as the unit of analysis. Each study was coded according to the presence or absence of each theme. A single study could be assigned to more than one category when it addressed multiple relevant dimensions. Therefore, the frequencies represent the number of studies in which each theme was identified, rather than the number of textual mentions within the articles.

To ensure reproducibility, each study was recorded in a coding matrix that included bibliographic information, country or context, study design, population or sample, methodological approach, main findings and thematic codes. Each thematic code was operationally defined prior to final classification. For example, ‘structural and symbolic barriers’ included evidence relating to institutional constraints, gender stereotypes, patriarchal norms, educational inequality or limited access to resources, whilst ‘sociocultural facilitating factors’ included evidence relating to role models, family or community support, gender-sensitive policies and empowerment strategies. The final themes were consolidated by merging overlapping codes and retaining those that recurred across the reviewed studies and aligned directly with the research questions.

Although specialised qualitative data analysis software such as ATLAS.ti or NVivo is commonly recommended for coding and thematic synthesis in systematic literature reviews, the present study adopted a structured manual coding approach supported by spreadsheet-based tools. Specifically, pivot tables were used not only for data organisation but also to facilitate systematic categorisation, comparison across studies, and the identification of thematic patterns.

To enhance transparency and replicability, the coding process followed a predefined analytical matrix. Each study was reviewed and coded according to bibliographic information, methodological approach, context, main themes, identified barriers, transformation strategies, leadership dimensions, and key findings. The coding categories were initially defined based on the research questions and were refined iteratively during the review process. Two researchers participated in validating the coding structure, and disagreements were resolved through discussion and consensus. Therefore, although specialised software such as ATLAS.ti or NVivo might have facilitated the management of qualitative data, the structured, spreadsheet-based approach proved appropriate for the objectives of this review, which focused on the systematic comparison of published studies rather than the analysis of primary qualitative interviews or raw textual data.

This approach ensured analytical rigour by applying predefined coding criteria, consistent classification procedures, and iterative validation of categories throughout the review process. Moreover, the use of spreadsheet-based analysis enabled transparent data handling, traceability of coding decisions, and reproducibility of results, as all transformations and categorisations were explicitly documented.

It is important to note that previous systematic reviews have successfully employed similar approaches when dealing with structured bibliographic data, particularly in studies focused on thematic classification and comparative analysis. Therefore, while advanced qualitative software may enhance analytical depth in certain contexts, the selected methodology is appropriate for this study's objectives and ensures sufficient rigour, transparency, and replicability.

### 2.7 Risk of bias

The risk of bias was assessed by identifying methodological limitations and potential publication biases in the selected studies. This was achieved by considering transparency of procedures, diversity of sources, geographical balance, and clarity of result presentation. This process enabled the determination of the level of reliability of the analysed evidence. The process incorporated a qualitative evaluation criterion to assess the methodological and theoretical quality of the articles, classifying them as high, medium, or low in reliability based on consistency and analytical rigour. The potential biases arising from the use of specific databases, the selection of keywords, and limitations in the reporting of results were also identified, as these factors can affect the representativeness of the final corpus. The results are summarised in Figure 1, corresponding to the PRISMA 2020 flow diagram.

## 3. Results

The results were organised according to the five research questions in order to comprehensively examine cultural barriers, transformative factors, and the dynamics of women’s leadership in rural settings. The analysis of the empirical and theoretical evidence collected allowed for the identification of common patterns across regions, along with contrasts stemming from the social, institutional and cultural conditions of each context. Emerging trends indicate a shift in the leadership of rural women, despite structural and symbolic constraints that limit their participation. Table 2 provides a synopsis of the selected studies that underpinned the comparative analysis and interpretation of the findings.

**Table 2.** Studies included in the research. Prepared by the author using data from Scopus and Web of Science.

Title	Authors (Year)	Main Themes	Methodology	Sample/Context	Key Findings/Conclusions
Analyzing the role of gender in entrepreneurship education and economic success in developing nations: the case of Colombia	de la Puente Pacheco et al., (2025)	Gender in entrepreneurship education and business success	Quantitative (Chi-square, t-tests, Regression analysis)	89 participants (Barranquilla, Colombia)	Gender-specific differences in program access; positive relationship between education and business success for both genders.
Creating Shared Value in Banking by Offering Entrepreneurship Education to Female Entrepreneurs	Taskin S et al., (2023)	Creating Shared Value (CSV) in female-centered banking and entrepreneurship education	Qualitative (Case study)	Female entrepreneurs (City Alo, Bangladesh)	Entrepreneurship education fosters client prosperity and addresses socio-economic challenges in women’s entrepreneurship.
Does entrepreneurship education in China develop entrepreneurial intention? the role of self-efficacy and experience	Xu J et al., (2023)	Entrepreneurship education, self-efficacy, and entrepreneurial intention	Quantitative (Structural Equation Modeling)	243 participants (China, female = 40.3%)	Entrepreneurship education has a significant positive impact on entrepreneurial intention; self-efficacy mediates this effect.
Entrepreneurship education and entrepreneurial intention: Do female students benefit?	Westhead and Solesvik (2016)	Links between entrepreneurship education, alertness, risk-taking, and gender	Quantitative (Hierarchical regression)	Business and engineering students (Unspecified university)	Female students in entrepreneurship education report lower levels of entrepreneurial intention, but those who cite alertness skills have higher intentions.
Entrepreneurship education in TVET institutions and entrepreneurial intentions of female students in Ghana: the social support factor	Padi A et al., (2022)	Impact of TVET entrepreneurship education on female students’ intentions	Mixed-methods (Quantitative survey + Interviews)	376 female students (Ghana)	Entrepreneurship education positively influences female students’ entrepreneurial intentions, especially when supported by social networks.
Evaluating the Role of Entrepreneurship Education in Shaping Female College Students’ Career Choices in China: A Multidimensional Analysis	Sun SL et al., (2025)	Gender, entrepreneurship education, and career choices	Quantitative (Regression analysis)	24,508 female students (China, across 31 provinces)	Courses and competitions are critical for female students' entrepreneurial career choices; policy initiatives have a limited effect.
Financial Literacy as a Key to Entrepreneurship Education: A Multi-Case Study Exploring Diversity and Inclusion	Medina-Vidal A et al., (2023)	Financial literacy and its impact on entrepreneurial education	Mixed-methods (Focus groups + Questionnaires)	Young Mexican students (17–24 years old, five cities)	Financial literacy correlates with better financial behaviours; critical thinking enhances financial decision-making, reducing the gender gap.
Gender Roles in Entrepreneurship Education to Social Entrepreneurial Intentions in Vietnam	Lan et al. (2023)	Gender roles in social entrepreneurship education and intentions	Quantitative (Partial least squares SEM)	811 students (Vietnam)	Significant gender differences in social entrepreneurship intentions; entrepreneurship education has varying effects by gender.
Gendered Entrepreneurship Education and the Fear of Failure	Guelich, U. (2022)	Gendered entrepreneurship education and the fear of failure	Quantitative (Survey + SEM)	1,668 entrepreneurs (Thailand)	Fear of failure impacts innovation, but knowing other entrepreneurs reduces its effect, especially for women.
Impact of Innovation and Entrepreneurship Education in a University Under Personality Psychology Education Concept on Talent Training and Cultural Diversity of New Entrepreneurs	Li et al., (2021)	Innovation and entrepreneurship education in universities	Mixed-methods (Surveys + Interviews)	University students (China, different majors)	Innovation dimension scores are higher among students, with female students showing slightly higher challenge spirit; social and cultural diversity also influence entrepreneurship outcomes.
Improving the Entrepreneurial Competence of College Social Entrepreneurs: Digital Government Building, Entrepreneurship Education, and Entrepreneurial Cognition	Xiang et al. (2022)	Entrepreneurship education, digital government, and gender differences	Quantitative (Regression analysis)	20,134 female students (31 provinces, China)	EE and digital government building positively influence female social entrepreneurs’ competence; gender differences are observed.
Overcoming Gender Gaps in Entrepreneurship Education and Training	Pimpa, N. (2021)	Financial literacy and its impact on entrepreneurship education	Mixed-methods (Focus groups + Questionnaires)	17–24-year-old students (Mexico, five cities)	Critical thinking positively correlates with financial behaviour; financial literacy reduces the gender gap in financial decision-making.
The effect of entrepreneurship education on the entrepreneurial intention of different college students: Gender, household registration, school type, and poverty status	Deng & Wang (2023)	Gender roles in social entrepreneurship education and social entrepreneurship intention	Quantitative (Partial least squares SEM)	811 students (Vietnam)	Significant gender differences in social entrepreneurship intentions; EE’s impact on gender is unclear.
The Impact of Entrepreneurial Self-Efficacy and Entrepreneurship on Entrepreneurial Intention: Entrepreneurial Attitude as a Mediator and Entrepreneurship Education Having a Moderate Effect	Ye & Kang (2025)	Gendered entrepreneurship education and the fear of failure	Quantitative (Survey + SEM)	1,668 entrepreneurs (Thailand)	Fear of failure impacts innovation, but knowing other entrepreneurs reduces its effect, especially for women.
Unveiling the role of entrepreneurship education on green entrepreneurial intentions among business students: gender as a moderator	Makuya & Changalima (2024)	Impact of entrepreneurship education on green entrepreneurial intentions	Quantitative (PLS-SEM)	204 business students (Tanzania)	EE positively impacts green entrepreneurial intentions; gender does not moderate this relationship despite stronger effects in males.
College students’ entrepreneurship education path and management strategy of start-up enterprises using causal attribution theory	Liu et al., (2025)	Entrepreneurship education, digital government, and gender differences	Quantitative (Regression analysis)	20,134 female students (31 provinces, China)	EE and digital government building positively influence female social entrepreneurs’ competence, with gender differences observed.
Educators and students in entrepreneurship education are challenging the “think entrepreneur–think male” paradigm	Stoker et al., (2025)	Gender issues in entrepreneurship education	Qualitative (Interviews)	32 students and educators (Netherlands)	Gendered constructs in EE are changing; the “think entrepreneur–think male” paradigm is being challenged.
Entrepreneurial, economic, and social well-being outcomes from an RCT of a youth entrepreneurship education intervention among native American adolescents	Tingey et al., (2020)	Entrepreneurship education, economic empowerment, and social well-being	Quantitative (RCT) + Qualitative (Focus groups)	394 Native American youth (USA)	Significant improvements in entrepreneurship knowledge and economic confidence among Native American youth; strong impact on social well-being.
Entrepreneurship Education among University Students as a Predictor of Female Entrepreneurial Undertakings	Vukmirović, V. (2019)	Female entrepreneurship and education	Conceptual (Literature review)	Female entrepreneurs (Global)	Gender-specific barriers to female entrepreneurship: a conceptual model to address them.
Entrepreneurship education and disability: An experience at a Spanish university	Muñoz et al., (2019)	Entrepreneurship education for disabled students	Quantitative (ANOVA)	50 students (Spain, with and without disabilities)	No significant differences between disabled and non-disabled students in terms of entrepreneurial attitudes after EE.
Entrepreneurship Education for Women—European Policy Examples of Neoliberal Feminism?	Berggren, C (2020)	Neoliberal feminism, gender in entrepreneurship education	Policy analysis (Neoliberal feminism perspective)	European Union policies (2004–2018)	The focus of EE policies is more about ideological education than promoting women’s self-employment.
Exploration and Practice of Maker Education Mode in Innovation and Entrepreneurship Education	Yang, Y. (2020)	Impact of entrepreneurial psychological quality and maker education	Quantitative (Survey + SPSS analysis)	20,000+ students (China, different majors)	EE and maker education influence entrepreneurial competence; family economy, sports, and grades impact students’ innovation and entrepreneurship abilities.
Gender-sensitive vocational and entrepreneurship education: addressing poverty for Caribbean women	Bahaw et al., (2025)	Gendered poverty, entrepreneurship education in TVET	Qualitative (Literature review)	Women in the Caribbean	Integrating EE into TVET can empower women, but gender-sensitive practices are critical to reducing barriers and enhancing opportunities.
Generative artificial intelligence in entrepreneurship education enhances entrepreneurial intention through self-efficacy and university support	Xie, Y & Wang, S 2025	Generative AI in entrepreneurship education	Quantitative (SEM)	346 students (China)	Generative AI enhances entrepreneurial self-efficacy and intention, and university support strengthens these effects.
How does entrepreneurial curiosity stimulate new venture ideas among Chinese undergraduates? The mediating role of promotion focus and the moderating role of entrepreneurial education	Li, C & Hu, R (2025)	Entrepreneurial curiosity and new venture ideas	Quantitative (PLS-SEM)	650 undergraduates (China)	I-type entrepreneurial curiosity positively affects new venture ideas; entrepreneurial education moderates this effect.
Impact of attitude towards entrepreneurship education and role models on entrepreneurial intention	Amofah & Saladrigues (2022)	Entrepreneurial intention, role models, gender, and EE	Quantitative (SEM, Multi-group analysis)	216 students (Spain)	Gender differences in entrepreneurial intention: the relationship between PSE and entrepreneurial competence is stronger for males.
Impact of personality traits and entrepreneurship education on entrepreneurial intentions of business and engineering students	Vodă & Florea (2019)	Personality traits, entrepreneurial education, and entrepreneurial intention	Quantitative (Multivariate logistic regression)	270 students (Romania)	Locus of control, need for achievement, and entrepreneurial education influence venture creation; males are more inclined towards entrepreneurship.
Role modeling as a pedagogical strategy in entrepreneurship education for women and girls: An interactive model of transformational learning	Oppedisano & Laird (2006)	Role modelling in entrepreneurship education for women	Qualitative (Interviews, case study)	Undergraduate students (US, multidisciplinary course)	Role models positively influence female students’ perceptions of entrepreneurship and their career aspirations.
Teacher-student empathic relationship shaping: an elimination mechanism for psychological segmentations in entrepreneurship education	Yi et al., (2025)	Teacher-student empathy, psychological segmentation, and EE	Quantitative (Experiments, SEM)	424 students (China)	Empathic teacher-student relationships enhance entrepreneurial confidence; cognitive and affective empathy play significant roles.
Teaching through television: Experimental evidence on entrepreneurship education in Tanzania	Bjorvatn et al., (2020)	Edutainment, entrepreneurship education, and school performance	Experimental (RCT, Field experiment)	2,000 secondary school students (Tanzania)	Edutainment shows increased interest in entrepreneurship, particularly among females, but has negative effects on school performance.
The analysis of the effect of entrepreneurship education, perceived desirability, and entrepreneurial self-efficacy on university students’ entrepreneurial intention	Suratno et al., (2019)	Entrepreneurship education, self-efficacy, perceived desirability, gender differences	Quantitative (SEM, Cross-sectional)	505 alumni (Jambi University, Indonesia)	EE positively affects entrepreneurial intention; gender differences were observed, with males having stronger self-efficacy.
The Development of Inclusive Agriculture Entrepreneurship Education Ecosystems for Young Entrepreneurs in Uganda	Bullock et al., (2025)	Inclusive agricultural entrepreneurship education in Uganda	Qualitative (Discourse analysis, interviews)	16 student entrepreneurs (Uganda)	Gender and financial literacy gaps remain barriers for women; EE should be more inclusive and support financial access.
The Effect of Entrepreneurship Education on Entrepreneurial Intention: Mediation of Entrepreneurial Self-Efficacy and Moderating Model of Psychological Capital	Wang et al., (2023)	Entrepreneurship education, self-efficacy, and psychological capital	Quantitative (SEM, Structural equation modelling)	757 students (Guangxi, China)	EE significantly impacts entrepreneurial intention; psychological capital moderates the effect of self-efficacy.
The Influence of Economic and Entrepreneurial Education on Perception and Attitudes towards Entrepreneurship	Ilieș et al., (2023)	Economic and entrepreneurial education, entrepreneurial intention	Quantitative (Regression analysis, PCA)	582 students (Romania)	EE and economic background significantly predict entrepreneurial intention, with students from economic backgrounds showing stronger intention.
The Re-integration of College Innovation and Entrepreneurship Education Under Young Entrepreneurs’ Enterprising Spirit and Professional Music Education	Qu et al., (2022)	Integration of innovation and entrepreneurship education with professional education	Quantitative (Survey, descriptive stats)	305 students (Music College, Xi’an, China)	Integration of IEE and professional education needs improvement; gender differences in entrepreneurship awareness.
The role of entrepreneurship education as a predictor of university students’ entrepreneurial intention	Zhang et al., (2014)	Entrepreneurship education, entrepreneurial intention, gender differences	Quantitative (Probit estimation)	494 students (10 universities, China)	Entrepreneurship education positively impacts entrepreneurial intention; gender, university type, and major significantly affect EI.
Unpacking Entrepreneurial Education: Learning Activities, Students’ Gender, and Attitude Toward Entrepreneurship	Padilla-Angulo et al., (2022)	Gender differences in entrepreneurial attitude and education	Quantitative (Survey, Post-hoc tests)	918 students (French business school)	Gender differences in entrepreneurial attitude; specific academic activities can boost EPA, with gender and academic level influencing outcomes.

The quality assessment showed that the included studies demonstrated an overall acceptable level of methodological and conceptual reliability. Most empirical studies were classified as having high reliability, as they featured clear objectives, appropriate quantitative or qualitative designs, consistent analytical procedures, and transparent conclusions. These studies included structural equation modelling, regression analysis, experimental or quasi-experimental approaches, qualitative interviews, discourse analysis and systematic coding procedures.

A smaller group of studies was classified as having moderate reliability. These studies were generally conceptual, pedagogical, policy-oriented, or descriptive in nature, or had limitations related to sample size, a restricted institutional context, limited methodological detail, or a lack of empirical validation. Nevertheless, they were retained because they provided relevant conceptual, pedagogical, or contextual information for understanding women’s entrepreneurship education, gender-sensitive training, role models, cultural barriers, and inclusive entrepreneurial ecosystems.

No study was excluded solely on the basis of the quality assessment. Instead, the assessment served as a basis for interpreting the evidence. Empirical studies with the highest scores were used as primary evidence in the synthesis, whilst those with medium scores were used as supplementary support, particularly when they provided conceptual explanations, contextual nuances, or pedagogical implications. This procedure helped to ensure that the conclusions and recommendations derived from the review were based on the strongest available evidence, whilst recognising the value of contextual and conceptual contributions.

It is important to clarify that the review did not exclusively include studies carried out in rural settings. Given the limited availability of studies that directly address entrepreneurship education for rural women, the body of research also incorporated studies on women’s entrepreneurship education, entrepreneurial intention from a gender perspective, leadership, empowerment and educational transformation in comparable educational and institutional contexts. These studies were included where their findings provided transferable insights into the cultural, institutional or gender-related mechanisms that may influence women’s entrepreneurship education in rural or structurally unequal settings.

The results are presented in Figure 2, which illustrates the distribution of the primary typologies of female leadership identified in the review. Community leadership is predominant, followed by inclusive, innovative, empowerment-centred, and self-efficacy-oriented approaches, while educational leadership is less frequent. This trend underscores the value of collaborative and transformational approaches in female leadership processes linked to education and social participation.

The results presented in Figure 3 demonstrate the distribution of the primary sociocultural factors that promote women’s entrepreneurial education. The role of entrepreneurial education, the empowerment of individuals through education, and the influence of role models are foundational, followed by family support, gender equality initiatives, and community networks. Collectively, these factors contribute to the creation of an environment conducive to the development of women’s leadership. The findings underscore the pivotal role of academic training, social support, and the visibility of role models in enhancing women’s entrepreneurial skills.

The results presented in Figure 4 illustrate the distribution of the primary structural and symbolic barriers that condition women’s participation in entrepreneurial education. The prevalence of institutional barriers is particularly salient, followed by issues of educational inequality, cultural stereotypes, and disparities in access to innovation. The influence of gender bias and patriarchal norms is identified to a lesser extent, yet these factors continue to exert their influence in educational and business spheres, thereby reflecting the persistence of inequalities that hinder the development of women’s entrepreneurial leadership.

The results presented in Figure 5 illustrate the distribution of the primary documented community impacts associated with women’s entrepreneurship education. It is evident that entrepreneurial self-efficacy and women’s economic empowerment are of particular significance, in addition to the enhancement of community resilience, local economic growth, and academic inclusion. These elements signify substantial progress in capacity building and opportunities. Social cohesion, an indicator of the consolidation of collective bonds and the positive impact of women’s participation in entrepreneurial ecosystems, is identified to a lesser extent.

The results presented in Figure 6 illustrate the distribution of the primary programmes and strengthening approaches identified in women’s entrepreneurship education. Gender-focused education initiatives are of particular note, followed by inclusive policy designs and experiential learning approaches, which are notable for their applicability in academic and social settings. Evidence suggests that actions aimed at fostering collaboration between universities and industry are in evidence, as are leadership and microfinance training programmes. These measures contribute to reducing structural gaps and promoting women’s participation in entrepreneurship.

The results were organised according to the research questions and allowed for a comprehensive understanding of the dimensions that comprise women’s entrepreneurial education. The analysis revealed the interaction of structural, symbolic, and community factors that influence women’s leadership and participation, along with programs aimed at strengthening their capacities.

## 4. Discussion

The findings of this study can be interpreted in light of existing theoretical frameworks on entrepreneurial education and intention. In line with previous research, the results confirm that entrepreneurial education is strongly influenced by contextual and cultural factors, which shape both learning processes and entrepreneurial outcomes ( Barba-Sánchez & Atienza-Sahuquillo, 2018; Cardella et al., 2024).

From a theoretical perspective, these results are consistent with the Theory of Planned Behavior, which highlights the role of individual perceptions, attitudes, and social norms in shaping entrepreneurial intention. In this study, cultural barriers and gender-related norms appear to function as contextual constraints that influence these cognitive and behavioral mechanisms.

Furthermore, the findings extend prior literature by emphasizing the role of cultural dynamics as a structuring dimension in entrepreneurial education. While previous studies have focused on psychological or institutional factors, this research integrates cultural barriers, perceptions, and transformation strategies into a unified framework, offering a more holistic understanding of entrepreneurial education processes.

Additionally, the results align with emerging research on gender and entrepreneurship, which suggests that deeply embedded social roles continue to influence women's entrepreneurial pathways ( Atienza-Barba et al., 2025). This highlights the need for educational models that incorporate gender-sensitive and culturally aware approaches to foster more inclusive entrepreneurial ecosystems.

The discussion is organised holistically in order to interpret the study’s findings in relation to the theoretical framework and the overall research objective. First, the results are analyzed, highlighting their relevance in the context of women’s entrepreneurial education. Subsequently, comparisons are made with national and international research, identifying both similarities and differences. In the following section, a conceptual framework is presented, derived from the results obtained. This section also includes a discussion of the theoretical, political, and practical implications. The study's limitations are then described, and future research directions are proposed to further explore the topic.

### 4.1 Results analysis

The analysis of the results indicates that women’s leadership is characterised by a foundation in collaborative, transformational, and inclusive approaches that promote active participation and empowerment in educational and social contexts. This leadership programme has been shown to enhance critical thinking skills, encourage innovation, and foster self-management, all of which are vital to reducing the structural inequalities that persist in the entrepreneurial sphere. In accordance with extant research, entrepreneurial education has been demonstrated to promote gender equality and critical thinking in decision-making ( Medina-Vidal et al., 2023), in addition to fostering the development of innovative capacities in culturally diverse environments ( Li et al., 2021).

The analysis of the results indicates that sociocultural factors play a pivotal role in women’s entrepreneurial education, promoting empowerment, training, and social support as fundamental elements for enhancing women’s participation. The provision of entrepreneurial education has been demonstrated to engender heightened self-confidence and to foster gender equality through the facilitation of inclusive and collaborative learning environments. These results align with research demonstrating the positive influence of entrepreneurship education on entrepreneurial intent and the creation of initiatives with social impact ( Lan et al., 2023), as well as on the adoption of sustainable and equitable practices ( Makuya & Changalima, 2024).

The analysis of the results indicates that the primary constraints on women’s engagement in entrepreneurship education are rooted in institutional barriers, educational disparities, and cultural stereotypes that impede access and innovation. The findings of this study demonstrate that social structures and patriarchal norms perpetuate gender disparities in education and leadership. In accordance with recent studies, entrepreneurship education requires the implementation of diversified strategies and inclusive policies with the aim of overcoming structural biases and strengthening women’s self-efficacy ( Sun et al., 2025; Wang et al., 2023). Such measures are considered to be conducive to the promotion of equitable and sustainable educational environments.

The analysis of the results indicates that the provision of education in entrepreneurship for female members of the community has a significant impact on the community as a whole, with the effects of this being the strengthening of self-efficacy, economic empowerment, and collective resilience. The aforementioned effects are reflected in local growth, academic inclusion, and the establishment of social support networks. The findings align with research demonstrating that entrepreneurship education promotes economic and social well-being in vulnerable communities ( Tingey et al., 2020) and fosters the development of sustainable entrepreneurial intentions in students through university education and role models ( Amofah & Saladrigues, 2022), consolidating equitable and participatory ecosystems.

The analysis of the results indicates that programmes and approaches for the enhancement of entrepreneurship education for women prioritise the incorporation of a gender perspective, inclusive policies, and experiential learning. These strategies have been shown to encourage women’s participation, strengthen their self-confidence, and promote practical training in academic and business contexts. The findings align with research showing that entrepreneurial education increases entrepreneurial intention, especially among women and students from urban areas ( Deng & Wang, 2023), and strengthens self-efficacy as a determining factor in the decision to become entrepreneurs ( Ye & Kang, 2025), thus consolidating equitable and transformative educational environments.

### 4.2 Comparison of results with other studies

The present study corroborates earlier research by underscoring the impact of structural, symbolic, and community factors on the evolution of female leadership. Moreover, it extends the existing discourse by incorporating entrepreneurial education as a transformative component of social and economic advancement. In contrast to the findings of Wulandari and Ahmad (2025), who emphasised digital and technological access disparities, this analysis focuses on institutional and cultural impediments. In contrast to the focus of Aljohani and Alharbi (2025) on financial self-efficacy, this study emphasises collaborative learning and educational equity. Moreover, it builds upon the contributions of Djatmiko et al. (2025) on digital inclusion by underscoring the pivotal role of social support and community networks in women’s empowerment. In contrast to the analysis of technological innovation by Nazir et al. (2025), this study prioritises education and cultural transformation as cornerstones of sustainability. In accordance with the findings of Deng et al. (2025), it acknowledges the significance of perceived capabilities and gender equity, incorporating a comprehensive educational dimension that links self-efficacy with community leadership.

### 4.3 Proposed conceptual framework

**Figure 7.**
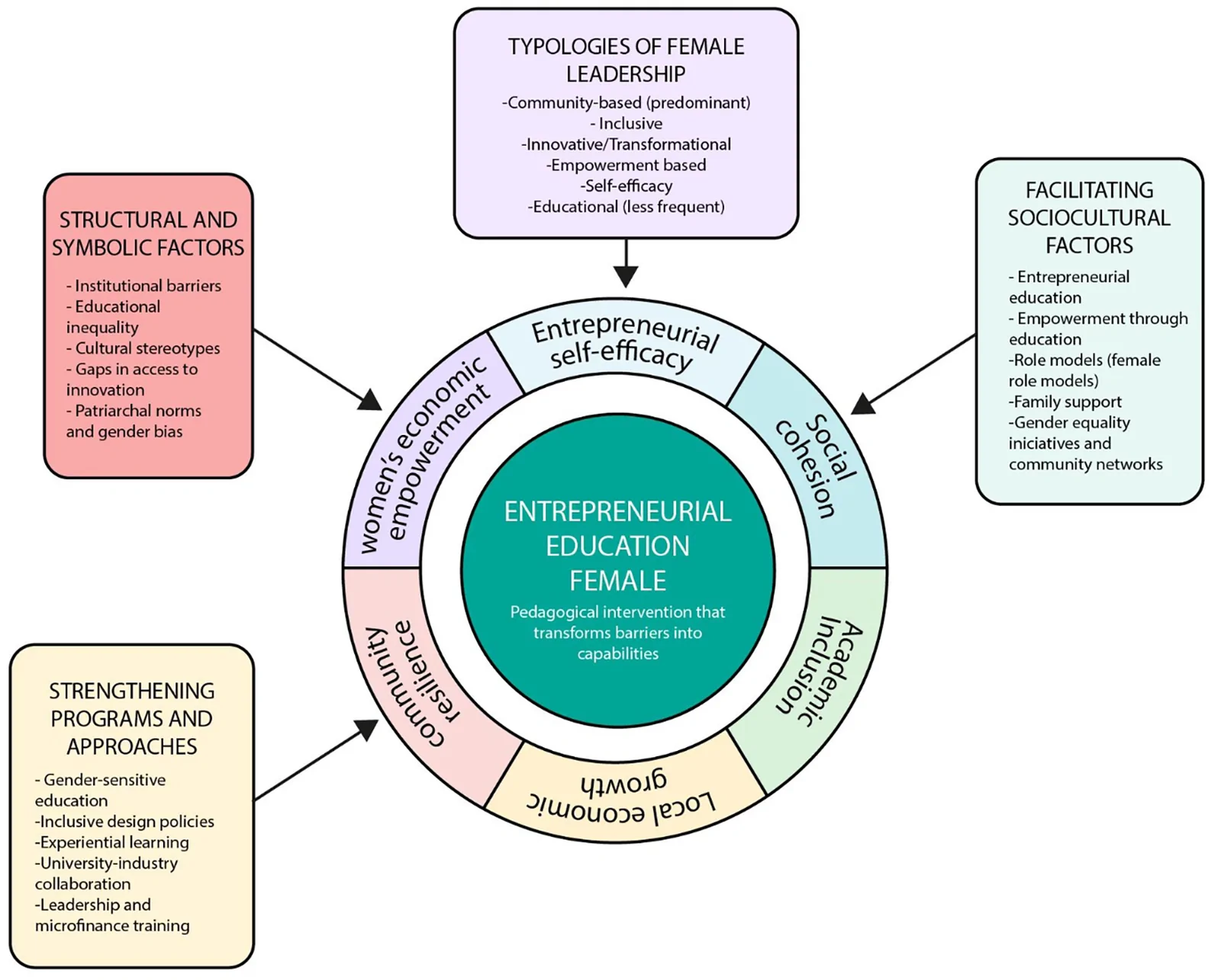
Conceptual framework of female entrepreneurial education. Prepared by the author based on the research results.

As illustrated in Figure 7, the proposed conceptual model integrates the determining factors of women’s entrepreneurial education. The framework under discussion articulates the structural, symbolic, sociocultural, and empowerment dimensions, reflecting their interaction in shaping women’s leadership. Entrepreneurial education is presented as a mediating force that transforms institutional and cultural barriers into opportunities for empowerment, equity, and sustainability, driving a continuous process of social transformation based on training, innovation, and community collaboration.

Building upon the systematic literature review, this study proposes a set of conceptual propositions that articulate the relationships between the key dimensions identified in female entrepreneurial education. These propositions are grounded in the qualitative synthesis of prior research and aim to provide a structured theoretical basis for the proposed conceptual framework. The conceptual framework is complemented by a set of propositions (P1–P5), which are visually represented in the model to illustrate the relationships between the identified dimensions.

P1 Cultural barriers negatively affect women's participation and engagement in entrepreneurial education.

P2 Individual perceptions, including self-efficacy, motivation, and perceived social norms, mediate the relationship between cultural context and entrepreneurial intention among women.

P3 Entrepreneurial education programs that incorporate gender-sensitive and context-aware approaches positively influence women's entrepreneurial competencies and intentions.

P4 Institutional and educational transformation strategies help reduce cultural barriers and foster inclusive entrepreneurial ecosystems.

P5 The integration of cultural awareness into entrepreneurial education enhances learning outcomes and supports women's entrepreneurial development.

These propositions clarify the conceptual logic of the proposed framework and show how the dimensions identified in the systematic review are interrelated. They also provide a theoretical basis for future empirical studies that may analyze the relationships among cultural barriers, women’s perceptions, educational strategies, institutional support, and empowerment outcomes.

### 4.4 Implications

***4.4.1 Theoretical Implications***

This study contributes to the literature on entrepreneurial education by addressing critical gaps in the understanding of cultural, gender, and institutional barriers that shape women’s entrepreneurial intentions. Previous research has often focused on individual psychological factors or institutional constraints in isolation, but this study integrates these elements into a unified framework. The findings extend the Theory of Planned Behavior by emphasizing the role of cultural and gendered social norms in mediating the influence of entrepreneurial education on women’s intentions. Furthermore, this research contributes to the growing body of literature on gender and entrepreneurship, highlighting how education can serve as a transformative force to challenge entrenched cultural biases and promote gender equality in entrepreneurial ecosystems.

***4.4.2 Methodological Implications***

From a methodological perspective, this study introduces a novel approach to the analysis of entrepreneurial education by combining qualitative data synthesis with a conceptual framework that integrates cultural and gender-related factors. The use of structured thematic coding, despite not relying on specialised software, ensured transparency, replicability, and rigorous analysis. This study demonstrates that manual coding combined with systematic pivot table analysis is a valid methodological approach for mapping complex educational phenomena, and can be replicated in other contexts, particularly in studies involving gender-sensitive education. Additionally, integrating cultural perspectives into the study of entrepreneurship education offers a valuable methodological tool for researchers interested in exploring the intersection of education, culture, and gender.

***4.4.3 Practical Implications***

The practical implications of this study are significant for educational policymakers, institutions, and practitioners aiming to foster more inclusive entrepreneurial ecosystems. The findings suggest that women’s participation in entrepreneurial education can be enhanced through gender-sensitive curricula and inclusive policies that address both institutional and cultural barriers. This study also highlights the importance of integrating collaborative learning, experiential education, and social support networks into entrepreneurial programs. These insights provide actionable recommendations for universities, particularly in regions with significant gender disparities, to create educational environments that empower women and equip them with the skills and confidence necessary for entrepreneurial success. Furthermore, the findings underscore the need for institutional reforms that promote gender equity and cultural transformation, offering valuable guidance for stakeholders in both the educational and entrepreneurial sectors.

### 4.5 Limitations and possible lines of future research

While this study makes significant contributions to understanding women’s entrepreneurial education, several limitations warrant consideration. First, the study’s focus on university students limits the generalizability of its findings to broader populations, particularly to early-stage entrepreneurial ventures or to individuals with prior entrepreneurial experience. Future research could address this gap by including participants from various entrepreneurial stages, such as nascent entrepreneurs or women leading established businesses, to provide a more comprehensive view of the factors influencing entrepreneurial intentions.

Second, while this study examines cultural and gender barriers, it does not fully explore their intersection with other dimensions, such as socioeconomic status, ethnic background, or geographic location. Future studies could integrate these additional variables to examine how multiple social identities shape women’s engagement in entrepreneurial education and practice. The inclusion of diverse populations could help uncover more nuanced insights into the structural challenges faced by different groups of women in entrepreneurial contexts.

Third, although the study employs a robust methodological approach, it relies on self-reported data, which can be subject to biases such as social desirability or respondent interpretation. Future research could complement these findings with mixed-methods approaches, such as qualitative interviews or ethnographic studies, to deepen understanding of women’s lived experiences in entrepreneurial education. This would allow for more in-depth insights into how women navigate educational environments and overcome barriers in practice.

Another limitation lies in the heterogeneity of the studies reviewed. Whilst the manuscript places particular emphasis on rural women and rural leadership, not all of the included studies were conducted in rural contexts. Several articles focused on university students, entrepreneurship training programmes or entrepreneurial intentions in non-rural settings. Consequently, conclusions regarding rural contexts should be interpreted with caution. Future research should include a greater number of studies focusing directly on rural women entrepreneurs, rural leadership and territorial development in Latin America and other underserved regions.

Finally, while the study identifies several strategies for enhancing women’s entrepreneurial education, it does not evaluate the effectiveness of these interventions in real-world settings. Future work could involve longitudinal studies or pilot programs to assess the impact of gender-sensitive and culturally inclusive entrepreneurial education programs on women’s entrepreneurial outcomes. By implementing and evaluating these interventions, researchers can gain a better understanding of how educational reforms can lead to lasting changes in women’s entrepreneurial participation and success.

## 5. Conclusions

The findings of this study should be interpreted as a synthesis of the evidence on women’s entrepreneurship education in various educational, institutional and socio-cultural contexts, with specific implications for rural and structurally unequal environments. Whilst some studies focus directly on rural women and community leadership, others provide indirect but relevant evidence on gender barriers, entrepreneurial intent, empowerment and educational strategies. Taken together, the results suggest that entrepreneurship education can serve as a strategic space for social transformation by reducing gender inequalities, strengthening leadership capacities and promoting inclusive innovation. However, its impact depends on the interplay between educational practices, cultural norms, institutional support and opportunities for women’s participation.

The analysis highlights the need to redefine models of entrepreneurship education from an equity and cultural-sensitivity perspective, and to position women as agents of change within educational, productive, and community ecosystems. This change requires challenging the narratives that perpetuate the exclusion of women and promoting collaborative networks between institutions, communities and productive sectors that strengthen their autonomy, resilience and leadership. Therefore, women’s entrepreneurship education should be understood not only as preparation for setting up businesses, but also as a collective process aimed at creating equitable, sustainable and inclusive environments where leadership becomes a tool for social innovation and cultural transformation.

## Ethical considerations

Ethical approval and Consent were not required.

## Data Availability

This study is based on source records retrieved from the Scopus and Web of Science databases, together with screening logs, data extraction tables, and PRISMA documentation generated throughout the review process. These materials support the identification, selection, and synthesis of the studies included in the analysis and ensure transparency in each methodological stage, from the initial search to the final thematic interpretation. The source records from Scopus and Web of Science are subject to database licensing and copyright restrictions and therefore cannot be considered fully open or reusable datasets under an open license. Although these records were cleaned, coded, and analyzed by the authors, their original source remains protected by the terms of use of the respective databases. For this reason, the full datasets cannot be redistributed freely but are shared exclusively for transparency and verification purposes. No individual, sensitive, or personal data were collected at any stage of this research, and the study did not involve human participants, interventions, or clinical data. Consequently, ethical approval or review by an Institutional Review Board (IRB) or equivalent ethics committee was not required. All procedures were conducted in accordance with international standards for research integrity and responsible data management. Aggregated screening counts, the PRISMA 2020 flow diagram, and the completed PRISMA 2020 checklist are publicly available in the Zenodo repository at https://doi.org/10.5281/zenodo.18625254 ( Valencia-Arias et al., 2025). Data are available under the terms of the Creative Commons Attribution 4.0 International license (CC-BY 4.0).
