# Supplementary material for: Cultural dynamics in entrepreneurial education: Barriers, perceptions, and transformation pathways
Source: F1000Res. 2026 Aug 3;15:315. Originally published 2026 Feb 24. [Version 3] doi: 10.12688/f1000research.178134.3 (PMC13562930; doi:10.12688/f1000research.178134.3)
Supplement: Peer review history

# Reviewer report: Reviewer response for version 3

**DOI:** 10.5256/f1000research.206414.r508146

**Published:** 2026-08-31

**Authors:** Louise Kelly

**Affiliations:** 1 University of La Verne, La Verne, CA, USA

This systematic review examines cultural, structural, and institutional barriers affecting women’s entrepreneurial education and leadership, with particular attention to rural and structurally unequal contexts. This is an important topic, and the manuscript makes a useful effort to bring together literature on entrepreneurship education, gender, leadership, empowerment, and culture that is often treated separately. The research questions are clearly stated, and the use of PRISMA, independent review by two researchers, quality appraisal, and structured thematic coding provide a reasonable foundation for the review.

 My primary concern is the alignment between the stated scope of the review and the evidence that is ultimately used to support its conclusions.

 The manuscript places considerable emphasis on rural women and, more specifically, rural Latin American contexts. However, the authors acknowledge that a substantial portion of the included literature is not based on rural populations and includes studies of university students and other educational and institutional populations across multiple regions. This does not invalidate the review, but it does require greater precision in how the findings are presented. Evidence drawn directly from rural women should be distinguished from evidence being extrapolated from other populations and contexts. The authors should also avoid making generalized statements about rural women when the underlying evidence does not consistently come from rural samples.

 A second issue concerns the movement from 129 studies included in the qualitative synthesis to 45 studies included in the detailed analysis. The manuscript needs to explain much more clearly how these 45 studies were selected. What criteria distinguished them from the remaining studies? This is particularly important if the figures, thematic frequencies, conceptual framework, or major conclusions are based primarily on this subset. A reader should be able to understand and replicate this stage of the review.

 The search strategy is clearly described for Scopus and Web of Science, but it is relatively narrow given the breadth of the research questions. Culture, rurality, leadership, and empowerment were not included as independent search blocks and instead entered primarily through eligibility decisions, manual searches, and thematic coding. The authors explain their reasoning, but this approach creates the possibility that relevant studies not explicitly framed as entrepreneurship education were missed. At minimum, this should be acknowledged more directly as a limitation.

 The quality appraisal is a useful addition, although the authors use a tailored five-item assessment rather than established appraisal tools for the various study designs represented in the review. Given the heterogeneity of the literature, I understand the rationale for developing a common framework. However, the authors should provide greater transparency about its application, including whether the assessments were independently conducted by two reviewers, how disagreements were resolved, and how many studies were classified as high, medium, and low quality.

 Similarly, the section labeled “risk of bias” is quite general. It identifies potential database, publication, keyword, and geographic biases but does not appear to constitute a formal study-level risk-of-bias assessment. I would either strengthen this section by explaining the actual procedure and results or relabel it to reflect more accurately what was assessed.

 There are also statements in the Limitations section that need correction. The manuscript refers to “the study’s focus on university students” and states that the study “relies on self-reported data.” Neither accurately describes this systematic review, which synthesizes studies using multiple populations and methodologies. These statements read as though they refer to a primary empirical study and should be rewritten as limitations of the evidence base included in this review.

 I also recommend greater caution in the conceptual framework. The manuscript describes entrepreneurial education as a “mediating” mechanism and at several points uses language suggesting causal relationships. Mediation is a specific empirical relationship that has not been tested in this review. The framework is potentially useful, but these relationships should be presented as conceptual relationships suggested by the literature rather than demonstrated causal or mediating mechanisms.

 No meta-analysis or inferential statistical analysis is conducted, so I regard the statistical analysis question as not applicable. The descriptive frequencies used in the thematic synthesis are appropriate provided they are clearly understood as frequencies of coded themes across studies rather than estimates of prevalence or effect size.

 Finally, there is an apparent inconsistency regarding funding that should be corrected. The manuscript states that no grants were involved in supporting the work, while the Acknowledgments state that the project was funded by CONCYTEC through the PROCIENCIA program under a specific contract.

 Overall, I find the manuscript relevant and potentially valuable. I particularly appreciate the attempt to connect entrepreneurship education with broader cultural, gender, and leadership dynamics. My reservations are primarily about methodological transparency and conceptual precision rather than the value of the review itself. Clarifying the rural and geographic scope, explaining the selection of the 45 studies used for detailed analysis, correcting the Limitations section, clarifying the risk-of-bias assessment, and moderating the causal and mediation language would substantially strengthen the manuscript.

Are the rationale for, and objectives of, the Systematic Review clearly stated?

Yes

Is the statistical analysis and its interpretation appropriate?

Not applicable

If this is a Living Systematic Review, is the ‘living’ method appropriate and is the search schedule clearly defined and justified? (‘Living Systematic Review’ or a variation of this term should be included in the title.)

Not applicable

Are sufficient details of the methods and analysis provided to allow replication by others?

Partly

Are the conclusions drawn adequately supported by the results presented in the review?

Partly

Reviewer Expertise:

Management, leadership, strategy, and entrepreneurial, leadership and strategy, innovation.

I confirm that I have read this submission and believe that I have an appropriate level of expertise to confirm that it is of an acceptable scientific standard, however I have significant reservations, as outlined above.

# Reviewer report: Reviewer response for version 3

**DOI:** 10.5256/f1000research.206414.r507412

**Published:** 2026-08-06

**Authors:** Teresa Felgueira

**Affiliations:** 1 Polytechnic Institute of Guarda, Guarda, Portugal

The authors have responded constructively to my previous review comments and have made substantial revisions to the manuscript. The revised version provides greater methodological transparency, particularly regarding the search strategy, quality assessment, and thematic synthesis procedures. The conceptual framework has been strengthened, and the discussion and conclusions are now better aligned with the evidence presented. These revisions have significantly improved the scientific rigor, clarity, and overall quality of the manuscript. I have no further substantive comments and consider that my previous concerns have been satisfactorily addressed.

Are the rationale for, and objectives of, the Systematic Review clearly stated?

Yes

Is the statistical analysis and its interpretation appropriate?

Not applicable

If this is a Living Systematic Review, is the ‘living’ method appropriate and is the search schedule clearly defined and justified? (‘Living Systematic Review’ or a variation of this term should be included in the title.)

Not applicable

Are sufficient details of the methods and analysis provided to allow replication by others?

Partly

Are the conclusions drawn adequately supported by the results presented in the review?

Partly

Reviewer Expertise:

Entrepreneurship; Entrepreneurial Orientation;  Entrepreneurship Education; Female Entrepreneurship; Market Orientation; Tourism.

I confirm that I have read this submission and believe that I have an appropriate level of expertise to confirm that it is of an acceptable scientific standard.

# Reviewer report: Reviewer response for version 2

**DOI:** 10.5256/f1000research.198053.r475632

**Published:** 2026-07-20

**Authors:** Virginia Barba-Sánchez

**Affiliations:** 1 Univ Castilla-La Mancha, Albacete, Spain

The revised manuscript has improved substantially compared to the previous version. The authors have made a significant effort to address many of the concerns raised in the earlier review, resulting in a clearer and more coherent paper. Nevertheless, there are still some issues that should be addressed before indexation.

 First, the title does not seem to fully reflect the actual content and scope of the paper. In particular, I would appreciate further justification for the removal of the term *“women”* from the previous version of the title. This change appears inconsistent with the focus outlined in the abstract, where concepts such as *gender equality* and *rural contexts* are explicitly highlighted. Moreover, the manuscript is presented as a systematic literature review, yet this methodological aspect is not sufficiently evident in the current title. I encourage the authors to reconsider the title so that it more accurately captures the central themes, population of interest, and methodological approach of the study.

 Second, in Section 4.3, each proposition should be justified independently and more explicitly. At present, the rationale supporting the propositions is not always clearly differentiated. To strengthen the theoretical contribution and improve the logical consistency of the manuscript, each proposition should be accompanied by its own specific argumentation, grounded in the evidence and literature discussed in the review.

 Overall, the manuscript has progressed considerably and is now much stronger than the previous version. Addressing the above issues would further enhance its clarity, coherence, and academic contribution.

Are the rationale for, and objectives of, the Systematic Review clearly stated?

Partly

Is the statistical analysis and its interpretation appropriate?

Partly

If this is a Living Systematic Review, is the ‘living’ method appropriate and is the search schedule clearly defined and justified? (‘Living Systematic Review’ or a variation of this term should be included in the title.)

No

Are sufficient details of the methods and analysis provided to allow replication by others?

Partly

Are the conclusions drawn adequately supported by the results presented in the review?

Partly

Reviewer Expertise:

Entrepreneurship; entrepreneurial education

I confirm that I have read this submission and believe that I have an appropriate level of expertise to confirm that it is of an acceptable scientific standard, however I have significant reservations, as outlined above.

# Reviewer report: Reviewer response for version 2

**DOI:** 10.5256/f1000research.198053.r490024

**Published:** 2026-06-17

**Authors:** Teresa Felgueira

**Affiliations:** 1 Polytechnic Institute of Guarda, Guarda, Portugal

Thank you for the opportunity to review the manuscript ‘Cultural dynamics in entrepreneurial education: Barriers, perceptions, and transformation pathways’ submitted to F1000Research. This manuscript presents a systematic review examining cultural barriers, perceptions, and transformation pathways in women's entrepreneurial education. The review focuses on factors influencing female participation, leadership, empowerment, and entrepreneurial development, while proposing a conceptual framework that integrates cultural, institutional, and educational dimensions.

 The topic is relevant and timely, particularly given the increasing interest in gender equality, entrepreneurship education, and inclusive economic development. The manuscript follows PRISMA 2020 guidelines and includes a substantial body of literature retrieved from Scopus and Web of Science.  The article is generally well organized and clearly written. However, several methodological and interpretative issues should be addressed before the review can be considered fully robust and reproducible.

 1.The introduction clearly explains the importance of women's entrepreneurial education and identifies a knowledge gap regarding the interaction between culture, leadership, and entrepreneurship education. The research objective is explicitly stated, and the review questions are clearly formulated. The rationale for conducting the review is adequately justified and supported by recent literature.

 2.The authors provide a reasonably detailed description of databases used. However, several aspects remain insufficiently described. The reported search strings focus primarily on "entrepreneurial education": "women"; "female".  Yet the review addresses broader themes such as leadership, cultural barriers, empowerment, social transformation, rural development. **The authors should explain how these concepts were captured through the search strategy or consider expanding the search terms**.

 The manuscript mentions that studies were classified according to reliability and methodological rigor, but no formal assessment tool is reported. **The authors should specify **which quality appraisal framework was used; assessment criteria; scoring procedures; results of the quality assessment. A summary table would strengthen transparency.

 Although coding and categorization are described, it remains nuclear how themes emerged; whether coding was performed independently by multiple reviewers; how disagreements were resolved; how frequencies reported in the figures were calculated.

 3. The overall conclusions are broadly consistent with the themes identified in the reviewed literature. However, some conclusions appear stronger than the evidence presented. The manuscript repeatedly emphasizes rural women, rural leadership, rural Latin American contexts. Nevertheless, many of the studies included in Table 1 focus on university students; entrepreneurship education programs; entrepreneurial intentions; non-rural settings.

 The authors **should either moderate conclusions** specifically related to rural contexts or **provide clearer evidence** demonstrating that the included literature adequately supports these claims.

 The proposed framework is interesting and potentially useful. However, the manuscript **would benefit from a clearer explanation** of how the propositions (P1–P5) emerged from the review findings rather than from theoretical interpretation alone.

**In conclusion, the following points should be addressed to strengthen the scientific rigor of the review**:

1. Provide a stronger justification for the search strategy and explain how key concepts such as culture, leadership, and empowerment were captured.
2. Describe in detail the quality assessment process and provide results for included studies.
3. Expand the description of the thematic coding and synthesis procedures to improve reproducibility.
4. Clarify how the frequencies shown in Figures 2–6 was generated.
5. Ensure that conclusions regarding rural women and rural leadership are fully supported by the evidence reviewed.
6. Explain more explicitly how the conceptual framework and propositions were derived from the systematic review findings.

I hope you find the above comments useful, and I wish you the best of luck with developing the manuscript.

Are the rationale for, and objectives of, the Systematic Review clearly stated?

Yes

Is the statistical analysis and its interpretation appropriate?

Not applicable

If this is a Living Systematic Review, is the ‘living’ method appropriate and is the search schedule clearly defined and justified? (‘Living Systematic Review’ or a variation of this term should be included in the title.)

Not applicable

Are sufficient details of the methods and analysis provided to allow replication by others?

Partly

Are the conclusions drawn adequately supported by the results presented in the review?

Partly

Reviewer Expertise:

Entrepreneurship; Entrepreneurial Orientation;  Entrepreneurship Education; Female Entrepreneurship; Market Orientation; Tourism.

I confirm that I have read this submission and believe that I have an appropriate level of expertise to confirm that it is of an acceptable scientific standard, however I have significant reservations, as outlined above.

# Response

**Published:** 2026-07-14

**Authors:** JHOANY ALEJANDRO VALENCIA ARIAS

We thank the reviewer for their invaluable contributions to the article. Below, we respond point by point:

**Comment:** The authors provide a reasonably detailed description of databases used. However, several aspects remain insufficiently described. The reported search strings focus primarily on "entrepreneurial education": "women"; "female". Yet the review addresses broader themes such as leadership, cultural barriers, empowerment, social transformation, rural development. The authors should explain how these concepts were captured through the search strategy or consider expanding the search terms.

**Response:** We agreed that the manuscript needed to clarify how broader themes such as leadership, cultural barriers, empowerment, social transformation and rural development were addressed. Given that the original search terms focused on entrepreneurship education and terms relating to women, we revised the Search Strategy section to explain that these broader concepts were incorporated through the eligibility criteria, full-text screening, supplementary manual searches and thematic coding. We also added this point as a methodological limitation, acknowledging that future reviews could use broader search strings that include these conceptual clusters from the initial identification stage.

 _________________________

**Comment: **The manuscript mentions that studies were classified according to reliability and methodological rigor, but no formal assessment tool is reported. The authors should specify which quality appraisal framework was used; assessment criteria; scoring procedures; results of the quality assessment. A summary table would strengthen transparency.

**Response:** A new quality assessment procedure has been added to the methodology section. The revised manuscript now specifies the assessment framework, the five assessment criteria, the scoring procedure and the reliability levels used to classify the included studies. A summary table has also been added, detailing the quality score and reliability level for each study. The results of the assessment were used to distinguish between core empirical evidence and complementary conceptual or contextual support, thereby enhancing the transparency and rigour of the review.

 _________________________

**Comment:** Although coding and categorization are described, it remains unclear how themes emerged; whether coding was performed independently by multiple reviewers; how disagreements were resolved; how frequencies reported in the figures were calculated.

**Response:** We have revised the ‘Data Processing’ section to provide a clearer explanation of the coding and thematic synthesis procedure. The revised text now explains that the themes emerged through a combined deductive-inductive process, starting with categories derived from the research questions and subsequently refined through full-text analysis. We have also clarified that the coding process was validated by two researchers and that any discrepancies were resolved through discussion and consensus. Finally, we have specified how the frequencies shown in Figures 2 to 6 were calculated, using each study as a unit of analysis and coding the presence or absence of each theme in the reviewed literature.

 _________________________

**Comment:** The manuscript repeatedly emphasizes rural women, rural leadership, rural Latin American contexts. Nevertheless, many of the studies included in Table 1 focus on university students; entrepreneurship education programs; entrepreneurial intentions; non-rural settings. The authors should either moderate conclusions specifically related to rural contexts or provide clearer evidence demonstrating that the included literature adequately supports these claims.

**Response:** We have revised the manuscript to tone down specific claims relating to rural contexts. We clarify that the review includes both studies focusing directly on rural contexts and studies of comparable educational and institutional settings that provide relevant evidence on gender barriers, entrepreneurship training, empowerment and leadership. We have also adjusted the discussion, conclusions and limitations to make it clear that the findings should be interpreted as evidence on women’s entrepreneurship training in various contexts, with implications for rural and structurally unequal settings.

 _________________________

**Comment:** The proposed framework is interesting and potentially useful. However, the manuscript would benefit from a clearer explanation of how the propositions (P1–P5) emerged from the review findings rather than from theoretical interpretation alone.

**Response:** We have expanded the “Proposed Conceptual Framework” section by adding a set of conceptual propositions. These propositions clarify how cultural and symbolic barriers, perceptions of women, gender-sensitive business education, institutional and community support, and culturally sensitive educational strategies interact to shape women’s business education and leadership.

 _________________________

**Comment: **Provide a stronger justification for the search strategy and explain how key concepts such as culture, leadership, and empowerment were captured.

**Response:** We have revised the ‘Search Strategy’ section to better justify the search terms and clarify how broader concepts such as culture, leadership and empowerment were incorporated. The revised text now explains that the main search terms in the database focused on the central intersection of the review — entrepreneurship education and women’s participation — to ensure thematic accuracy. We have also clarified that broader concepts such as cultural barriers, leadership dynamics, empowerment, rural development and social transformation were incorporated through the eligibility criteria, full-text review, supplementary manual searches and thematic coding.

 _________________________

**Comment: **Describe in detail the quality assessment process and provide results for included studies.

**Response:** A new quality assessment procedure has been added to the methodology section. The revised manuscript now specifies the assessment framework, the five assessment criteria, the scoring procedure and the reliability levels used to classify the included studies. A summary table has also been added, detailing the quality score and reliability level for each study. The results of the assessment were used to distinguish between core empirical evidence and complementary conceptual or contextual support, thereby enhancing the transparency and rigour of the review.

 _________________________

**Comment:** Expand the description of the thematic coding and synthesis procedures to improve reproducibility.

**Response:** We have revised the ‘Data Processing’ section to expand on the description of the coding and thematic synthesis procedures. The revised version explains that the coding followed a combined deductive-inductive approach, beginning with categories derived from the research questions and subsequently refined through analysis of the full text. We have also clarified that the coding process was validated by two researchers, that discrepancies were resolved by consensus, and that the frequencies in Figures 2 to 6 were calculated using each study as the unit of analysis. Furthermore, we have added further details regarding the coding matrix, the operational definitions of the thematic categories and the consolidation of the final themes to improve reproducibility.

 _________________________

**Comment:** Clarify how the frequencies shown in Figures 2–6 was generated.

 Response: Aplica la respuesta anterior

 _________________________

**Comment:** Ensure that conclusions regarding rural women and rural leadership are fully supported by the evidence reviewed.

**Response:** We reviewed the manuscript to ensure that the conclusions regarding rural women and rural leadership are more closely aligned with the evidence analyzed. We amended the wording throughout the manuscript and clarified that the findings should be interpreted as evidence on women’s entrepreneurship education in various contexts, with specific implications for rural settings and those characterized by structural inequalities.

 _________________________

**Comment:** Explain more explicitly how the conceptual framework and propositions were derived from the systematic review findings.

**Response:** We have revised the conceptual framework section to clarify how the framework and propositions were derived from the findings of the systematic review. The revised text now explains that the propositions are based on the thematic synthesis of the reviewed literature and are linked to the main categories identified in the analysis.

 _________________________

# Reviewer report: Reviewer response for version 1

**DOI:** 10.5256/f1000research.196482.r462148

**Published:** 2026-03-18

**Authors:** Virginia Barba-Sánchez

**Affiliations:** 1 Univ Castilla-La Mancha, Albacete, Spain

Thank you for the opportunity to review the manuscript ‘Cultural barriers in women's entrepreneurial education: Challenges, perceptions and transformation strategies’ submitted to F1000Research. I enjoyed reading the manuscript and found it informative on entrepreneurial education.

 The manuscript has great potential, but the reasoning does not flow naturally. The paper must be improved in line with the comments below:

 1. The title is very descriptive but not overly wordy. It needs to be broader to better reflect the study's aims.

 2. While the authors provide purpose in the initial paragraphs, research gaps and significant contributions are not clear! How is this study different and unique?

 3. In the introduction, this study needs to be enhanced with more arguments exemplifying the importance of entrepreneurial education and this research. There is a lot of published work in the 2020s to strengthen the narrative on this, such as Barba-Sanchez et al. (refer to reference no. 1), Cardella et al. (refer to reference no. 2) or Atienza-Barba et al. (refer to reference no. 3).

 4. An article must be self-contained, i.e. it must contain all the information necessary for its comprehension. Table 1 currently lists only the title, authors, and year of each study. However, this information is already available in the reference list, so the table in its current form does not add substantial value to the review. It might be more informative to include elements such as the main themes addressed in each study, the methodology employed, the sample or context, and the key findings or conclusions. This would enrich comparative analysis and offer readers a clearer understanding of the contributions of each selected paper.

 5. In the methodology section, the manuscript mentions that ‘The information was organised into pivot tables that facilitated comparative analysis and the generation of descriptive graphs aimed at visualising trends.’ While tables can be useful for organizing information, it may have strengthened the rigor of the analysis to use specialized qualitative analysis software—such as ATLAS.ti or NVivo—to support coding, categorization, and thematic synthesis. Employing such tools is generally recommended in systematic literature reviews because they enhance transparency, replicability, and analytical depth. Please explain.

 6. Given that the authors propose a Conceptual framework of female entrepreneurial education, they should also identify the propositions that stem from the systematic literature review. This would reinforce the conceptual robustness and clarify how each proposition is grounded in the qualitative synthesis of prior research.

 7. The discussion needs improvement and lacks significant theoretical reference to the findings.

 8. Theoretical, methodological, and practical implications should be further elaborated and explicitly tied to the identified gaps and the novelty of the investigation.

 9. The authors should develop the limitations and future work. Please do not list the future research lines; integrate them into the text.

 I hope you find the above comments useful, and I wish you the best of luck with developing the paper further.

Are the rationale for, and objectives of, the Systematic Review clearly stated?

Partly

Is the statistical analysis and its interpretation appropriate?

Partly

If this is a Living Systematic Review, is the ‘living’ method appropriate and is the search schedule clearly defined and justified? (‘Living Systematic Review’ or a variation of this term should be included in the title.)

No

Are sufficient details of the methods and analysis provided to allow replication by others?

Partly

Are the conclusions drawn adequately supported by the results presented in the review?

Partly

Reviewer Expertise:

Entrepreneurship; entrepreneurial education

I confirm that I have read this submission and believe that I have an appropriate level of expertise to state that I do not consider it to be of an acceptable scientific standard, for reasons outlined above.

## References

[1] : Entrepreneurial intention among engineering students: The role of entrepreneurship education. *European Research on Management and Business Economics, 24(1), 53–61* .2018;

[2] : Analyzing university students’ entrepreneurial intentions under the influence of the COVID-19 pandemic. *International Entrepreneurship and Management Journal* .2024;20(4) : 10.1007/s11365-024-00945-x2567-2590 10.1007/s11365-024-00945-x

[3] : Gender differences in the impact of ecological awareness on entrepreneurial intent. *International Entrepreneurship and Management Journal* .2025;21(1) : 10.1007/s11365-025-01084-7 10.1007/s11365-025-01084-7

# Response

**Published:** 2026-03-24

**Authors:** JHOANY ALEJANDRO VALENCIA ARIAS

Thank you very much for your valuable feedback. We have addressed all of the recommendations made. Below is a point-by-point response:

 1. The title is very descriptive but not overly wordy. It needs to be broader to better reflect the study's aims.

**Response:** The title of the manuscript has been revised to broaden its conceptual scope and more fully reflect the study’s objectives, reducing its level of specificity and better aligning it with the article’s overall contribution.

 2. While the authors provide purpose in the initial paragraphs, research gaps and significant contributions are not clear! How is this study different and unique?

**Response:** A new paragraph has been added to the introduction that clearly outlines the research gaps identified in the literature, as well as the study’s theoretical and practical contributions. Furthermore, it emphasizes the integrative and multidimensional nature of the proposed approach, highlighting its unique value compared to previous studies.

 3. In the introduction, this study needs to be enhanced with more arguments exemplifying the importance of entrepreneurial education and this research. There is a lot of published work in the 2020s to strengthen the narrative on this, such as Barba-Sanchez et al. (refer to reference no. 1), Cardella et al. (refer to reference no. 2) or Atienza-Barba et al. (refer to reference no. 3).

**Response:** The introduction was strengthened by incorporating additional arguments and recent evidence that highlight the importance of entrepreneurship education. In particular, recent studies suggested by the reviewer (Barba-Sánchez et al., Cardella et al., Atienza-Barba et al.) were included to reinforce the theoretical framework and better contextualize the study’s relevance.

 4. An article must be self-contained, i.e. it must contain all the information necessary for its comprehension. Table 1 currently lists only the title, authors, and year of each study. However, this information is already available in the reference list, so the table in its current form does not add substantial value to the review. It might be more informative to include elements such as the main themes addressed in each study, the methodology employed, the sample or context, and the key findings or conclusions. This would enrich comparative analysis and offer readers a clearer understanding of the contributions of each selected paper.

**Response:** Table 1 has been revised and improved by including additional details, such as the main topics addressed in each study, the methodology used, the context or sample of the studies, and the key findings. This not only provides a more comprehensive overview of the selected studies but also enhances the comparative analysis and facilitates understanding of the contributions of each study.

 5. In the methodology section, the manuscript mentions that ‘The information was organized into pivot tables that facilitated comparative analysis and the generation of descriptive graphs aimed at visualizing trends.’ While tables can be useful for organizing information, they may have strengthened the rigor of the analysis to use specialized qualitative analysis software—such as ATLAS.ti or NVivo—to support coding, categorization, and thematic synthesis. Employing such tools is generally recommended in systematic literature reviews because they enhance transparency, replicability, and analytical depth. Please explain.

**Response:** The methodology section was expanded to justify the use of pivot tables as an analytical tool, explaining how systematic criteria for coding, categorization, and validation were applied. Furthermore, it is argued that this approach ensures the transparency, traceability, and replicability of the analysis, consistent with previous studies.

 6. Given that the authors propose a Conceptual framework of female entrepreneurial education, they should also identify the propositions that stem from the systematic literature review. This would reinforce the conceptual robustness and clarify how each proposition is grounded in the qualitative synthesis of prior research.

**Response:** Theoretical propositions derived from the systematic review were incorporated, which complement the conceptual framework and clarify the relationships among its dimensions.

 7. The discussion needs improvement and lacks significant theoretical reference to the findings.

**Response:** The discussion section was strengthened by incorporating a stronger theoretical foundation, integrating relevant conceptual frameworks, and establishing an explicit dialogue between the findings and the existing literature. In addition, the critical comparison with previous studies was improved, and the study’s theoretical contribution was clarified.

 8. Theoretical, methodological, and practical implications should be further elaborated and explicitly tied to the identified gaps and the novelty of the investigation.

**Response**: The theoretical, methodological, and practical implications were discussed in the discussion section, explicitly linking them to the identified research gaps and the novelty of the study. The theoretical implications contribute to the literature by integrating gender and cultural elements into existing models of entrepreneurial intention. At the methodological level, the approach used is justified, highlighting its applicability and replicability in other contexts. Finally, the practical implications offer clear recommendations for the implementation of inclusive educational strategies that promote female participation in entrepreneurship education.

 9. The authors should develop the limitations and future work. Please do not list the future research lines; integrate them into the text.

**Response:** The section on limitations and future research directions was strengthened by seamlessly integrating the identified limitations with the study’s future directions.

# Response

**Published:** 2026-07-14

**Authors:** JHOANY ALEJANDRO VALENCIA ARIAS

We thank the reviewer for their invaluable contributions to the article. Below, we respond point by point:

**Comment: **The title is very descriptive but not overly wordy. It needs to be broader to better reflect the study's aims.

**Response: **The title of the manuscript has been revised to broaden its conceptual scope and more fully reflect the study’s objectives, reducing its level of specificity and better aligning it with the article’s overall contribution.

 _________________________

**Comment:** While the authors provide purpose in the initial paragraphs, research gaps and significant contributions are not clear! How is this study different and unique?

**Response:** We revised the Introduction to make the research gaps and contributions

 _________________________

**Comment:** In the introduction, this study needs to be enhanced with more arguments exemplifying the importance of entrepreneurial education and this research. There is a lot of published work in the 2020s to strengthen the narrative on this, such as Barba-Sanchez et al. (refer to reference no. 1), Cardella et al. (refer to reference no. 2) or Atienza-Barba et al. (refer to reference no. 3).

**Response:** Thank you for this valuable suggestion. We have revised the Introduction to emphasize the importance of entrepreneurship education and the relevance of this study. Specifically, we have expanded and reformulated the analysis using recent literature, such as Barba-Sánchez and Atienza-Sahuquillo (2018), Cardella et al. (2024), Atienza-Barba et al. (2025), and added two new references: Passarelli and Bongiorno (2025) and Serpente et al. (2025). The revised version better explains the role of entrepreneurship education in the development of motivation, competencies, resilience, sustainability-oriented attitudes, gender perspectives, and ecosystem-based learning. We have also clarified the research gap by emphasizing the need for an integrative approach that connects cultural barriers, women’s perceptions, leadership dynamics, and pathways to transformation in rural and similar contexts.

 _________________________

**Comment:** An article must be self-contained, i.e. it must contain all the information necessary for its comprehension. Table 1 currently lists only the title, authors, and year of each study. However, this information is already available in the reference list, so the table in its current form does not add substantial value to the review. It might be more informative to include elements such as the main themes addressed in each study, the methodology employed, the sample or context, and the key findings or conclusions. This would enrich comparative analysis and offer readers a clearer understanding of the contributions of each selected paper.

**Response:** We revised Table 1 by adding the following columns: Main Themes, Methodology, Sample/Context, and Key Findings/Conclusions.

 _________________________

**Comment:** In the methodology section, the manuscript mentions that ‘The information was organized into pivot tables that facilitated comparative analysis and the generation of descriptive graphs aimed at visualizing trends.’ While tables can be useful for organizing information, it may have strengthened the rigor of the analysis to use specialized qualitative analysis software—such as ATLAS.ti or NVivo—to support coding, categorization, and thematic synthesis. Employing such tools is generally recommended in systematic literature reviews because they enhance transparency, replicability, and analytical depth. Please explain.

**Response:** We have revised the “Data Processing” section to better explain the analytical procedure used in the review. The revised text clarifies that the study adopted a structured manual coding approach, supported by spreadsheet tools, including pivot tables and a predefined analytical matrix. We also specify how the coding categories were defined, refined, and validated. While ATLAS.ti or NVivo might have facilitated the management of qualitative data, the spreadsheet-based approach proved adequate for this review, as it focused on the systematic comparison, categorization, and thematic synthesis of the published studies.

 _________________________

**Comment: **Given that the authors propose a Conceptual framework of female entrepreneurial education, they should also identify the propositions that stem from the systematic literature review. This would reinforce the conceptual robustness and clarify how each proposition is grounded in the qualitative synthesis of prior research.

**Response: **We have expanded the “Proposed Conceptual Framework” section by adding a set of conceptual propositions. These propositions clarify how cultural and symbolic barriers, perceptions of women, gender-sensitive business education, institutional and community support, and culturally sensitive educational strategies interact to shape women’s business education and leadership.

 _________________________

**Comment:** The discussion needs improvement and lacks significant theoretical reference to the findings.

**Response: **The discussion section was strengthened by incorporating a stronger theoretical foundation, integrating relevant conceptual frameworks, and establishing an explicit dialogue between the findings and the existing literature. In addition, the critical comparison with previous studies was improved, and the study’s theoretical contribution was clarified.

 _________________________

**Comment: **Theoretical, methodological, and practical implications should be further elaborated and explicitly tied to the identified gaps and the novelty of the investigation.

**Response:** The theoretical, methodological, and practical implications were discussed in the discussion section, explicitly linking them to the identified research gaps and the novelty of the study. The theoretical implications contribute to the literature by integrating gender and cultural elements into existing models of entrepreneurial intention. At the methodological level, the approach used is justified, highlighting its applicability and replicability in other contexts. Finally, the practical implications offer clear recommendations for the implementation of inclusive educational strategies that promote female participation in entrepreneurship education.

 _________________________

**Comment: **The authors should develop the limitations and future work. Please do not list the future research lines; integrate them into the text.

**Response:** The section on limitations and future research directions was strengthened by seamlessly integrating the identified limitations with the study’s future directions.

 _________________________
